# Testis-specific lncRNA *Teshl* regulates acrosome biogenesis to maintain sperm structure and function

**DOI:** 10.1186/s13578-026-01563-6

**Published:** 2026-03-30

**Authors:** Seung Pyo Hong, Seong Hyeon Hong, Gwidong Han, Seung Jae Lee, Youngsoo Oh, Chunghee Cho

**Affiliations:** 1 Department of Life Sciences, Gwangju Institute of Science and Technology, Gwangju, 61005 Republic of Korea; 2 RNA Therapeutics Institute, University of Massachusetts Chan Medical School, Worcester, MA 01605 USA; 3 KAIST AI-CRED Institute, Korea Advanced Institute of Science and Technology, Daejeon, 34051 Republic of Korea; 4 GIST Advanced Institute of Instrumental Analysis, Gwangju Institute of Science and Technology, Gwangju, 61005 Republic of Korea

**Keywords:** *Teshl*, Long noncoding RNA (lncRNA), Acrosome biogenesis, Spermiogenesis, Male fertility, Reproduction

## Abstract

**Background:**

The testis exhibits the highest tissue-specific gene expression of all organs, including both coding and non-coding genes. Our previous study reported that *Teshl*, a testis-specific long noncoding RNA, associates with heat shock factor 2 (HSF2), a transcription factor, to regulate Y chromosome gene expression and sperm fertility.

**Results:**

We conducted an in-depth analysis of the function and molecular mechanisms of *Teshl* using *Teshl*-knockout (KO) mice. Remarkably, the absence of *Teshl* caused abnormal acrosome biogenesis during spermiogenesis, resulting in malformed and disorganized acrosomes and abnormal sperm head morphology. *Teshl*-KO sperm also exhibited altered acrosome reaction and reduced motility. A thorough examination of all available relevant datasets identified three *Teshl*-related proteins associated with acrosome structure and function: actin related protein T1 (ACTRT1), acrosin binding protein (ACRBP) and dickkopf-like acrosomal protein 1 (DKKL1). Our findings suggest that these proteins are directly or indirectly regulated by *Teshl*-HSF2 through transcriptional or protein-interaction mechanisms.

**Conclusions:**

The present findings significantly expand our understanding of the important roles of *Teshl*, a pioneering testis-specific lncRNA, by elucidating its acrosomal function and mechanism in male reproduction.

**Supplementary Information:**

The online version contains supplementary material available at 10.1186/s13578-026-01563-6.

## Background

Mammalian spermatogenesis is an essential developmental process involving successive mitotic, meiotic, and post-meiotic phases (spermiogenesis) that is tightly regulated by gene expression programs [1]. The testis is distinctive for its rich expression of tissue-specific genes, including both coding and noncoding genes [2]. Understanding the function of such testis-specific genes is essential for understanding male reproduction.

Spermatogenesis leads to the production of highly specialized spermatozoa, which are structurally divided into a head and a tail, both of which are formed during the spermiogenic stage [3]. Although sperm head morphology varies considerably across species, mouse sperm are characterized by an oval, hook-shaped head – a distinctive feature of rodent sperm [4, 5]. The sperm head is composed of the nucleus and the acrosome, together with supporting structures, such as perinuclear theca (PT) and the acroplaxome [6]. The nucleus stores paternal genetic information, and as spermatogenic cells develop from round to elongating spermatids, their histones are replaced by transition proteins, which are in turn replaced by protamines, resulting in highly compacted chromatin that stabilizes the DNA [7]. This process ensures proper sperm head shape and preserves genomic integrity upon delivery to the egg [8, 9]. The acrosome arises from Golgi-derived vesicles in round spermatids and subsequently matures into a condensed structure that surrounds the nuclear surface [10]. This process occurs simultaneously with nuclear condensation. Although it does not contain genetic information, the acrosome is indispensable for fertilization, as it facilitates sperm penetration of the egg zona pellucida [11–13]. It is not surprising that the integrity of the sperm head – crucial for sperm function during fertilization – is regulated by testis-expressed genes.

Long noncoding RNAs (lncRNAs), defined as transcripts longer than 200 nucleotides with limited protein-coding potential, are increasingly considered important regulators of diverse biological processes, including transcription, translation, protein interaction, differentiation, metabolism, and signaling [14, 15]. A characteristic feature of lncRNAs is their tissue-specific expression, and no other organ has a more enriched tissue-specific lncRNA expression profile than the testis [2]. Nevertheless, only a limited number of testis-specific lncRNAs have been functionally characterized; thus, their diverse roles in spermatogenesis are largely unexplored [16–18].

The lncRNA, *Teshl* (testicular germ cell-specific HSF2-interacting lncRNA), is one of the best-characterized testis-specific lncRNAs. *Teshl* is spatiotemporally expressed in the nuclei of round and elongating spermatids and has been shown to interact with heat shock factor 2 (HSF2) to regulate transcription of the male-specific region of mouse Y chromosome long arm (MSYq)-linked genes, such as *Sly* (Sycp3-like Y-linked) and *Ssty* (spermiogenesis-specific transcript on the Y) [19]. Although *Teshl* has been characterized as a transcriptional regulator in the testis, its role in germ cell development – and whether it has a broader or more diverse molecular function – remains unknown. Analyses of *Teshl*-knockout (KO) mice have revealed a variety of phenotypes, including reduced litter size, a female-biased sex ratio, and marked defects in sperm head morphology. However, the molecular mechanisms underlying these phenotypes remain unclear. As a “pioneer” testicular lncRNA molecule, *Teshl*, particularly the nature of its regulatory mechanism, is of great importance in the field of non-coding gene research.

In this study, we conducted an in-depth investigation of the function of *Teshl* in testes, focusing on the most prominent abnormal phenotype observed in *Teshl*-KO mice: altered sperm head morphology. Using a comprehensive set of research methods, we revealed that *Teshl* is required for maintaining the structural stability of the acrosome and supporting the expression of multiple acrosomal proteins, providing insight into the unexpected and diverse molecular roles of *Teshl* in sperm function. Our findings imply that testis-specific lncRNAs act as critical regulators of sperm head development and function, providing a pioneering example of their importance in male reproduction.

## Materials and methods

### Animals

All animal experiments were performed in accordance with Korean Food and Drug Administration (KFDA) guidelines. Protocols were reviewed and approved by the Institutional Animal Care and Use Committee (IACUC) of Gwangju Institute of Science and Technology (GIST) (permit number: GIST-2024-032, GIST-2025-011). *Teshl-*KO mice used in this study were obtained by breeding mice generated in a previous study [19]. Testis samples were obtained from 8–10-week-old male mice, frozen in liquid nitrogen, and stored at -80 °C. Sperm samples were extracted from the epididymis of mice on the day of the experiment.

### In vitro fertilization (IVF)

Superovulation was induced in B6D2F1 female mice (8–10 weeks old) by injection of 7.5 international units (IU) of pregnant mare serum gonadotropin (PMSG; Prospec), followed 48 h later by injection of 7.5 IU of human chorionic gonadotropin (hCG; Prospec). Cumulus-oocyte complexes were collected from oviducts 15 h after hCG injection. Cumulus-enclosed oocytes were pre-incubated in human tubal fluid (HTF) medium (MilliporeSigma) at 37 °C in 5% CO_2_ for 1 h prior to sperm injection. Sperm were collected from 8–10-week-old wild type (WT) and *Teshl*-KO male mice and pre-incubated in HTF medium at 37 °C in 5% CO_2_ for 90 min to induce capacitation. Following pre-incubation, cumulus-enclosed oocytes were inseminated with 1× 10^6^ sperm cells and incubated in HTF medium at 37 °C in 5% CO_2_ for 8 h. After incubation, embryos were washed and cultured in potassium simplex optimized medium (KSOM). Two-cell, morula, and blastocyst embryos were collected at 48, 96 and 120 h post-hCG injection, respectively. For Hoechst staining, performed immediately after the 8-h incubation of sperm with oocytes, embryos were fixed in 3.7% formaldehyde (MilliporeSigma) in phosphate-buffered saline (PBS) containing 0.1% bovine serum albumin (BSA; Sigma-Aldrich) and permeabilized by incubating with 0.25% Triton X-100 in PBS containing 0.1% BSA. Embryos were then blocked with 5% BSA overnight at 4 °C. For nuclear staining, embryos were incubated with 1 µg/ml Hoechst 33,342 (Invitrogen) in 3% BSA containing 0.1% Triton X-100 for 30 min at room temperature. Sample images were obtained using a microscope (Leica Microsystem) equipped with an eXcope X9 camera (Dixi Science) using a 405 nm excitation laser and auto exposure at 10X magnification.

### Embryo PCR

Embryos were genotyped using a REDExtract-N-Amp PCR kit (MilliporeSigma) as described by the manufacturer. Briefly, blastocysts were collected 120 h after hCG injections, and individual embryos were transferred into a mixture of extraction solution and tissue preparation solution. Samples were incubated sequentially for cell lysis and DNA release according to the manufacturer’s protocol. The following primer pairs targeting the indicated regions were used for genotyping analysis: myogenin (*Myog*; autosomal gene), 5’- TTA CGT CCA TCG TGG ACA GC -3’ (forward) and 5’- TGG GCT GGG TGT TAG TCT TA -3’ (reverse); a Y chromosome-specific region, 5’CGGTGT TTG GCG TGA AAT GTCGG TGT TTG GCG TGA AAT GT -3’ (forward) and 5’ - AAC TGT TGT CCG TAG AGC CG -3’ (reverse). glyceraldehyde-3 phosphate dehydrogenase (*Gapdh*, internal control), 5’- TGT GTC CGT CGT GGA TCT GA -3’ (forward) and 5’- TTG CTG TTG AAG TCG CAG GAG -3’ (reverse). PCR reactions (10 µl) contained 1× REDExtract-N-Amp PCR ReadyMix, 1 µl of each primer, and 2 µl of template DNA. Cycling conditions were 94 °C for 5 min, followed by 32 cycles of 94 °C for 30 s, 58–60 °C for 30 s, and 72 °C for 30 s, with a final extension at 72 °C for 7 min. Amplified fragments were analyzed by agarose-gel electrophoresis.

### Nuclear morphology analysis

Mature sperm were collected from WT and *Teshl*-KO mice and counted using a hemocytometer under a light microscope. Sperm were spread onto a slide glass and allowed air to dry. For nuclear staining, cells were fixed with 4% paraformaldehyde (GeneAll), permeabilized with 0.5% Triton X-100, and blocked with 3% BSA (Sigma-Aldrich). After blocking, cells were stained with Hoechst dye (1 µg/ml; Invitrogen) for 30 min at room temperature. Sperm were analyzed under a fluorescence microscope (Leica Microsystems) equipped with a 405 nm excitation laser and an eXcope X9 camera (Dixi Science), with auto-exposure at 40X magnification. Hoechst images were analyzed using *Nuclear Morphology analysis software (version 2.1.0)*.

### Acrosome morphology analysis

Sperm were collected from WT and *Teshl*-KO mice and prepared as described for *Nuclear morphology analysis* (above). For dickkopf-like acrosomal protein 1 (DKKL1) staining, samples were incubated overnight at 4 °C with anti-DKKL1 primary antibodies (1 µg/ml; R&D Systems), followed by incubation with Alexa Fluor 594-conjugated secondary antibodies (1:200; Invitrogen) for 1 h at room temperature. For acrosome staining, mature sperm were stained with lectin-peanut agglutinin (lectin-PNA) (20 µg/m; Invitrogen) for 1 h at 4 °C. For nuclear staining, sperm were stained with Hoechst (1 µg/ml; Invitrogen) for 30 min at room temperature. DKKL1 immunoreactivity was imaged with an FV3000RS confocal laser-scanning microscope (Olympus), and acrosomes were imaged with a fluorescence microscope (Leica Microsystems). Paraffin-embedded sections of mouse testes were deparaffinized using a dewaxing solution (Elabscience) and rehydrated with a graded series of 100%, 95%, and 70% ethanol (MilliporeSigma). Heat-induced epitope retrieval was performed using pressure cooker at 110 °C for 10 min. After blocking with 3% BSA containing 0.03% Triton X-100 in Tris-buffered saline (TBST), samples were incubated with lectin-PNA (20 µg/ml; Invitrogen) for 1 h at 4 °C and Hoechst dye (1 µg/ml; Invitrogen) for 10 min at room temperature. Stained sections were observed under a fluorescence microscope (Leica Microsystems).

### Toluidine blue staining

Samples of mature sperm from WT and *Teshl*-KO mice, collected and counted as described above, were fixed with an ethanol: acetone (1:1) solution (MilliporeSigma) for 30 min at 4 °C, followed by hydrolysis in 0.1 N HCl for 5 min at 4 °C. After three washes with distilled water, samples were stained with 0.05% toluidine blue (Sigma-Aldrich), prepared in McIlvaine buffer (pH 3.5), for 10 min at room temperature. Stained sperm were analyzed under a light microscope (Leica Microsystems) at 40X magnification using auto-exposure with an eXcope X9 camera (Dixi Science).

### Scanning electron microscopy (SEM) and transmission electron microscopy (TEM) analyses

For SEM analyses, mature sperm were fixed with 4% paraformaldehyde (GeneAll) for 1 h at room temperature and washed three times with 1X PBS. Specimens were dehydrated with a graded series of 50%, 70%, 80%, 90%, 95% and 100% ethanol (MilliporeSigma); resuspended in a butanol: ethanol (1:1) mixture (Sigma-Aldrich/MilliporeSigma) for 15 min, and spread onto aluminum foil. Sperm were coated with platinum for 2 min under vacuum and subsequently examined using a Thermo Scientific Verios 5 XHR scanning electron microscope (Thermo Fisher Scientific) at an accelerating voltage of 5 kV. For TEM analyses, testes were fixed in 2.5% glutaraldehyde (Sigma-Aldrich) in 0.2 M cacodylate buffer overnight at 4 °C, cut into 1 × 1 mm^3^ section, post-fixed with 1% osmium tetroxide (Sigma-Aldrich) on ice for 2 h, and washed three times with 10X PBS. Samples were then dehydrated with a graded series of 70%, 80%, 90% and 100% ethanol and dehydrated again with a graded series of ethanol: propylene oxide (1:1) (MilliporeSigma/Sigma-Aldrich), propylene: eponate (2:1) (Sigma-Aldrich/PELCO) and propylene oxide: eponate (1:1) (PELCO) using a tissue processor (TP) (Leica). The samples were incubated in resin: eponate (1:1) mixture (PELCO) overnight in a 60 °C oven. Ultra-thin Sect.  (50 nm) were cut using an ultramicrotome (EM UC7/Leica), collected onto a TEM grid (Carbon Square Mesh, EMS), stained with uranyl citrate and lead citrate using an automatic contrasting instrument (EM AC20) (Leica), and observed using a transmission electron microscope (Tecnai F30) (Thermo Fisher Scientific).

### Acrosome reaction

Mature sperm were collected from WT and *Teshl*-KO mice, and ~ 5 × 10^6^ cells/ml were placed into a 400-µl drop of TYH medium and dispersed by gentle resuspension. Samples were incubated for 4 h at 37 °C in a humidified 5% CO_2_ environment. After incubation, 200 µl of sperm suspension was transferred to a new microtube. AR was induced by adding 20 mM A23187 (Sigma-Aldrich), after which samples were further incubated for 15 min at 37 °C in a 5% CO_2_ environment. Sperm were spread onto slide glass and allowed to air dry. As controls, non-reacted sperm were directly placed onto slide glasses without incubation and air dried. Cells were fixed with 4% paraformaldehyde (GeneAll), permeabilized with 0.5% Triton X-100, and blocked with 3% BSA (Sigma-Aldrich). After blocking, cells were incubated overnight at 4 °C with anti-izumo sperm-egg fusion 1 (IZUMO1) primary antibody (1:500; BioAcademia), followed by incubation with Alexa Fluor 647-conjugated secondary antibodies (1:200; Invitrogen) for 1 h at room temperature. After washing, cells were stained with lectin-PNA (20 µg/ml; Invitrogen) for 1 h at 4 °C. For nuclear staining, cells were stained with Hoechst (1 µg/ml; Invitrogen) for 30 min at room temperature. Samples were observed using a slide scanner (SLIDEVIEW VS200 system) (Olympus) with auto exposure at 40X magnification.

### Sperm motility analysis

Epididymal sperm, collected by dissecting the cauda epididymis, were incubated for 10 min at 37 °C. The sperm suspension was then transferred into a 150 µl drop of TYH medium, covered with paraffin oil, and incubated for 30 min at 37 °C in a 5% CO_2_ environment. After incubation, capacitated sperm were diluted 1:20 in fresh TYH medium. Motile sperm were recorded using a microscope (Leica Microsystems) equipped with an eXcope X9 camera (Dixi Science). Videos were preprocessed using ImageJ (National Institutes of Health) [20], and sperm motility parameters were analyzed in randomly selected fields using the OpenCASA system. All measurements were performed in both biological and technical triplicates.

### Quantitative RT-PCR

Total RNA was isolated from mouse testes using TRIzol reagent (Ambion). Complementary DNA (cDNA) was synthesized from DNase I-treated total RNA using an Omniscript RT kit (Qiagen) according to the manufacturer’s instructions. qRT-PCR was performed with TOPreal qPCR 2X premix (Enzynomics) using the following primer pairs: *Gapdh*, 5’- TGT GTC CGT CGT GGA TCT GA -3’ (forward) and 5’- TTG CTG TTG AAG TCG CAG GAG -3’ (reverse); *Dkkl1*, 5’- ACA ACT TCT TCT CCT CCC C -3’ (forward) and 5’- GTT GTC AGT CAC CTT GTC TAT C -3’ (reverse); actin-related protein T1 (*Actrt1*), 5’- GGA GAC CCT GAT GGT GCT AT -3’ (forward) and 5’- CAC ACA CCG AAT CCC CGA AG -3’ (reverse); acrosin-binding protein (*Acrbp*), 5’- GGA GAA TAC CTG CAC CAT GAC TC -3’ (forward) and 5’- TGT GTC GCC TTC CGA GAT TGT C -3’ (reverse). Each reaction was performed in triplicate. Relative gene expression levels were calculated using the 2^−ΔΔCt^ method [21] and normalized to *Gapdh* expression.

### Western blotting

Testis lysates were prepared by homogenizing testes in radioimmunoprecipitation assay (RIPA) buffer (Pierce) supplemented with a protease inhibitor cocktail. Protein concentrations were determined using a bicinchoninic acid assay (Pierce). Equal amounts of protein (30 µg/sample) were resolved by sodium dodecyl sulfate-polyacrylamide gel electrophoresis (SDS-PAGE) and transferred onto polyvinylidene difluoride (PVDF) membranes (Cytiva). Membranes were blocked with 5% nonfat milk for 1 h at room temperature and incubated overnight 4 °C with primary antibodies against the following proteins: ACTRT1 (1:10000; Abcam), actin-like 7 A (ACTL7A) (1:2000; Proteintech), ACTRT2 (1:2000; Proteintech), ACRBP (1:1000; St. John’s Laboratory), heat shock protein A2 (HSPA2) (1:5000; R&D Systems), heat shock protein 90, beta (Grp94), member 1 (HSP90B1) (1:2000; Abbexa), DKKL1 (0.5 µg/ml; R&D Systems), IZUMO1 (1:15000; BioAcademia), sperm equatorial segment protein 1 (SPESP1) (1:1000; BioAcademia), and α-tubulin (1:10000; MilliporeSigma). After washing with 1X TBST, membranes were incubated with the following horseradish peroxidase (HRP)-conjugated secondary antibodies (as appropriate): anti-rabbit lgG (1:5000–1:10000; Promega), anti-mouse lgG (1:10000; Santa Cruz Biotechnology), anti-goat lgG (1:10000; Santa Cruz Biotechnology) and anti-rat lgG (1:10000; Novus Biologicals). Immunoreactive signals were visualized, and band intensities were quantified using Image J software (National Institutes of Health).

### Statistical analysis

All experiments were conducted at least in triplicate for each sample. Data are presented as the means ± SEM. Data distribution did not show obvious deviation from normality, and parametric statistical analyses were therefore applied. For sperm motility analyses involving multiple parameters, results were interpreted based on consistency across related measures. After analysis of variance using an F-test, statistical analysis performed with Student’s *t*-test, and *P* values are indicated in the figure legends.

## Results

### Fertilization is impaired in sperm from *Teshl*-KO mice 

**Fig. 1:**
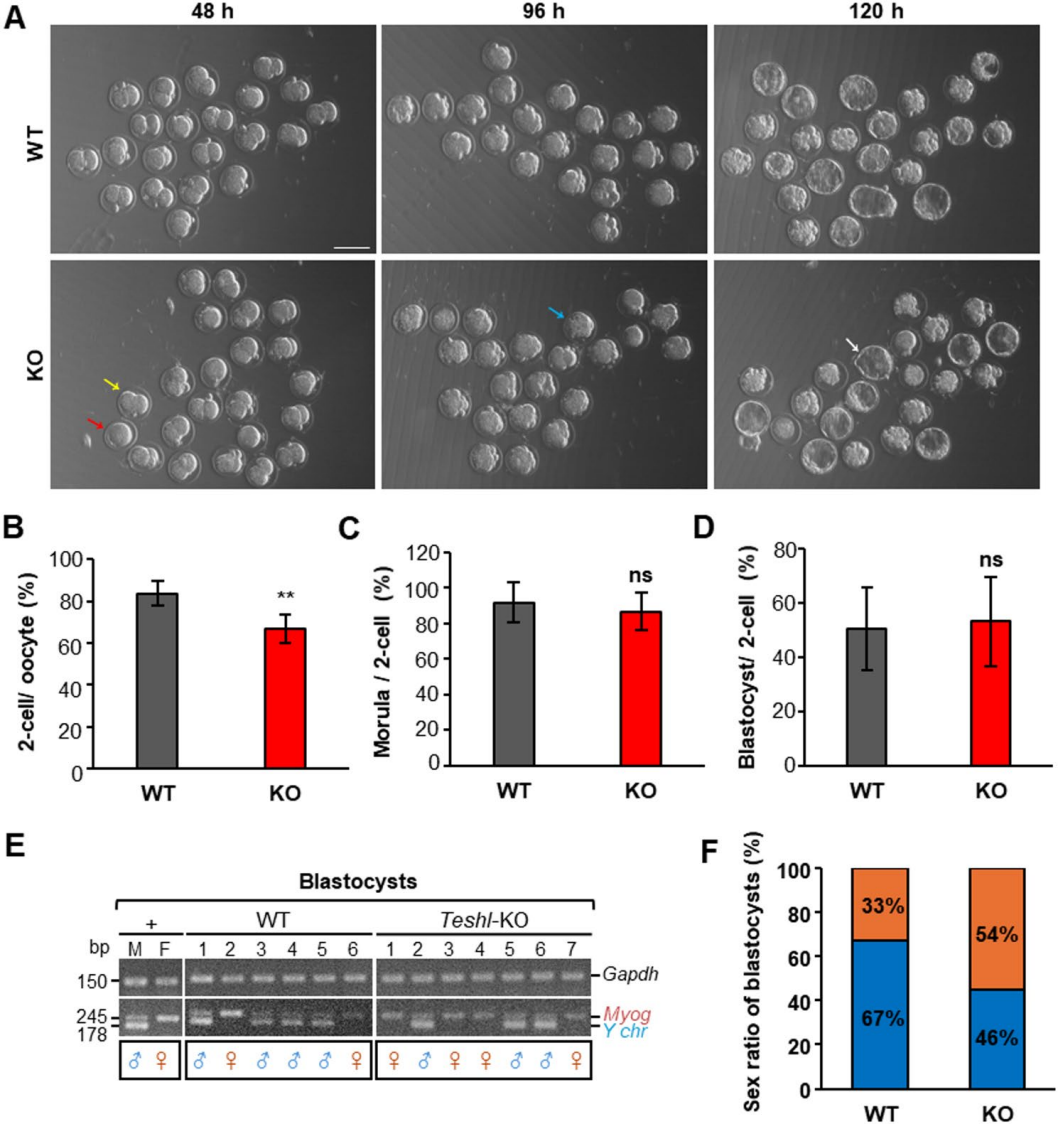
Assessment of fertilization efficiency and early embryogenesis in oocytes fertilized by *Teshl*-KO sperm. **A **Representative micrographs showing the developmental stages of embryos derived from WT and *Teshl*-KO sperm at 48, 96 and 120 h post hCG injection. Both fertilized and unfertilized eggs were observed at 48 h; morula-stage and blastocyst-stage embryos were observed at 96 and 120 h, respectively. Yellow arrow, fertilized egg; red arrow, unfertilized egg; blue arrow, morula; white arrow, blastocyst. Scale bar: 100 μm. **B**–**D** Bar graph showing the percentage of two-cell embryos obtained from retrieved oocytes (**B**), the percentage of two-cell embryos that progressed to the morula stage (**C**), and the percentage of two-cell embryos that developed into blastocysts (**D**), following in vitro fertilization of WT and *Teshl*-KO sperm. Data are presented as means ± SEM (*n* = 6; *P > 0.05, **P > 0.01, two-tailed Student’s *t* test). **E** Representative genomic PCR results for DNA extracted from individual blastocysts, used to determine embryonic sex ratios. Sex was determined by amplification of a Y chromosome-specific region, an autosomal gene (*Myog*) and *Gapdh*, used as a genomic DNA quality control marker. **F** Bar graph showing the sex ratio of blastocysts. Orange bar, female embryo; blue bar, male embryo. Embryos showing only the *Myog* band were classified as XX, whereas those showing both Y-specific and *Myog* bands were classified as XY. Data are presented as means ± SEM (*n* = 6; *P > 0.05, two-tailed Student’s *t* test)

Our previous study reported that male *Teshl*-KO mice produced a reduced number of offspring and a distorted sex ratio [19]. To determine the stage at which this fertility abnormality occurs, we conducted an in vitro fertilization analysis using WT and *Teshl*-KO male mice (Fig. 1A). Cumulus-enclosed oocytes were co-incubated with WT or *Teshl*-KO sperm, and the resulting eggs were retrieved, cultured, and monitored for embryonic development. The number of embryos that developed to the two-cell stage was significantly reduced in eggs fertilized by *Teshl*-KO sperm compared to WT sperm (Fig. 1B). An analysis of Hoechst-stained eggs also showed that the number of zygotes fertilized by *Teshl*-KO sperm was reduced compared to WT sperm (Supplementary Data 1 A–D). However, there was no significant difference in the number of embryos that progressed from the two-cell stage to morula and blastocyst stages between the two groups, suggesting that the cause of the decrease in fertility occurs at the gamete cell fertilization stage rather than during embryonic development (Fig. 1C, D; Supplementary Data 2 A). A polymerase chain reaction (PCR) analysis of blastocysts demonstrated a significant female-biased sex ratio in embryos derived from eggs fertilized by *Teshl*-KO sperm compared to WT sperm, consistent with a previous report [19] (Fig. 1E, F; Supplementary Data 2 B, C). These results show that the impaired fertility observed in *Teshl*-KO mice is attributable to a decrease in fertilization, strongly suggesting that abnormalities in the properties of *Teshl*-KO sperm are directly related to fertilization, not embryogenesis; that is, development is impaired owing to damaged sperm nuclei. The specific step of fertilization at which *Teshl*-KO sperm are impaired remains to be determined.

### A *Teshl* deficiency leads to sperm head abnormalities

**Fig. 2:**
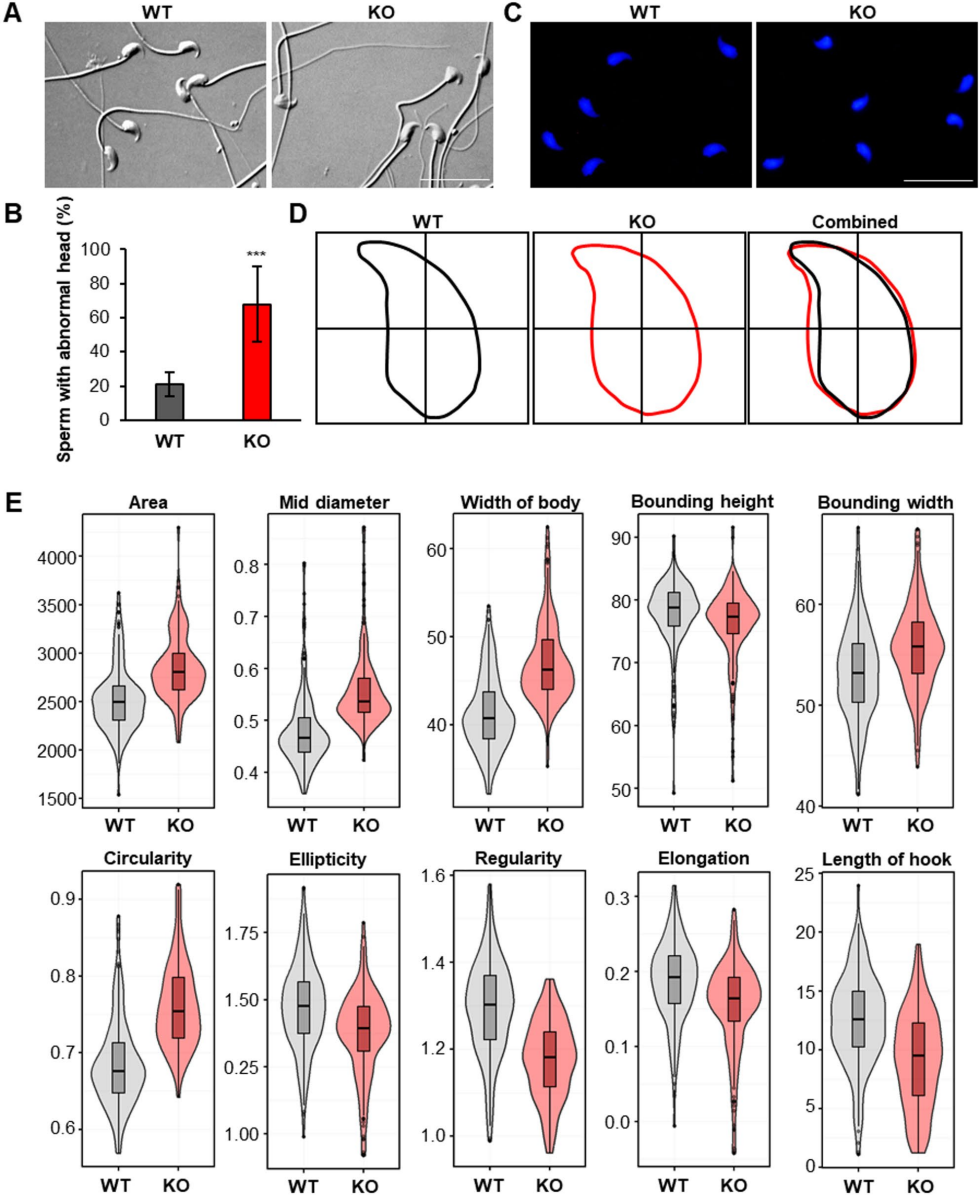
Morphological analysis of *Teshl*-KO mice sperm heads. **A** Representative bright-field images of WT and *Teshl*-KO sperm. **B** Bar graph showing the percentage of sperm with an abnormal head. Data are presented as means ± SEM (*n* = 3; ***P < 0.001, two-tailed Student’s *t* test). **C** Images of Hoechst-stained sperm from WT and *Teshl*-KO mice. Scale bar: 50 μm. **D** Overall head morphology of sperm from WT and *Teshl*-KO mice, together with an overlay image illustrating morphological differences. Black outlines, WT sperm head; red outlines, *Teshl*-KO sperm head. A total of 509 heads (blue) were analyzed (WT = 283; *Teshl*-KO = 226). **E** Various parameters of WT and *Teshl*-KO sperm nuclei. Data are presented as means ± SEM (*n* = 3; *P*-values were calculated using the non-parametric Mann-Whitney-Wilcoxon test)

To elucidate the basis of the decreased fertilizing capacity observed in *Teshl*-KO sperm, we focused on sperm properties that potentially influence fertilization. To this end, we performed an in-depth investigation of sperm head morphology, extending a brief, previous analysis of abnormal morphologies of *Teshl*-KO sperm [19]. Any change in size or shape compared with a normal sperm head was considered abnormal for purposes of this analysis. A quantitative, bright-field microscopic analysis of mature sperm collected from the cauda epididymis and vas deferens of WT and *Teshl*-KO mice (Fig. 2A; Supplementary Data 3 A, B) revealed a significant increase (~ 3.3-fold) in the proportion of sperm from *Teshl*-KO mice with abnormal head morphology (67%) compared with WT mice (20%) (Fig. 2B). To further characterize the sperm head morphological abnormalities observed in *Teshl*-KO mice, we used a nuclear morphology analysis tool [22] that quantifies the mean values of various parameters of nuclear shape across comparable sperm populations and allows analysis based on Hoechst-stained images (Fig. 2C). This analysis revealed that the nuclear morphology of *Teshl*-KO sperm was characterized by a more rounded and thicker head and a shorter and blunter apical hook compared with WT sperm (Fig. 2D). A statistical analysis of quantitative parameters – including area, mid-body diameter, maximum body width, bounding box height, bounding box width, circularity, ellipticity, regularity, elongation and hook length – confirmed the significance of these morphological abnormalities in *Teshl*-KO sperm compared with WT sperm (Fig. 2E; Supplementary Data 4). Our findings indicate that *Teshl* deficiency is associated with an abnormal sperm head morphology that is attributable, at least in part, to altered nuclear morphology.

### Aberrant sperm head morphology is associated with acrosomal deformities

**Fig. 3:**
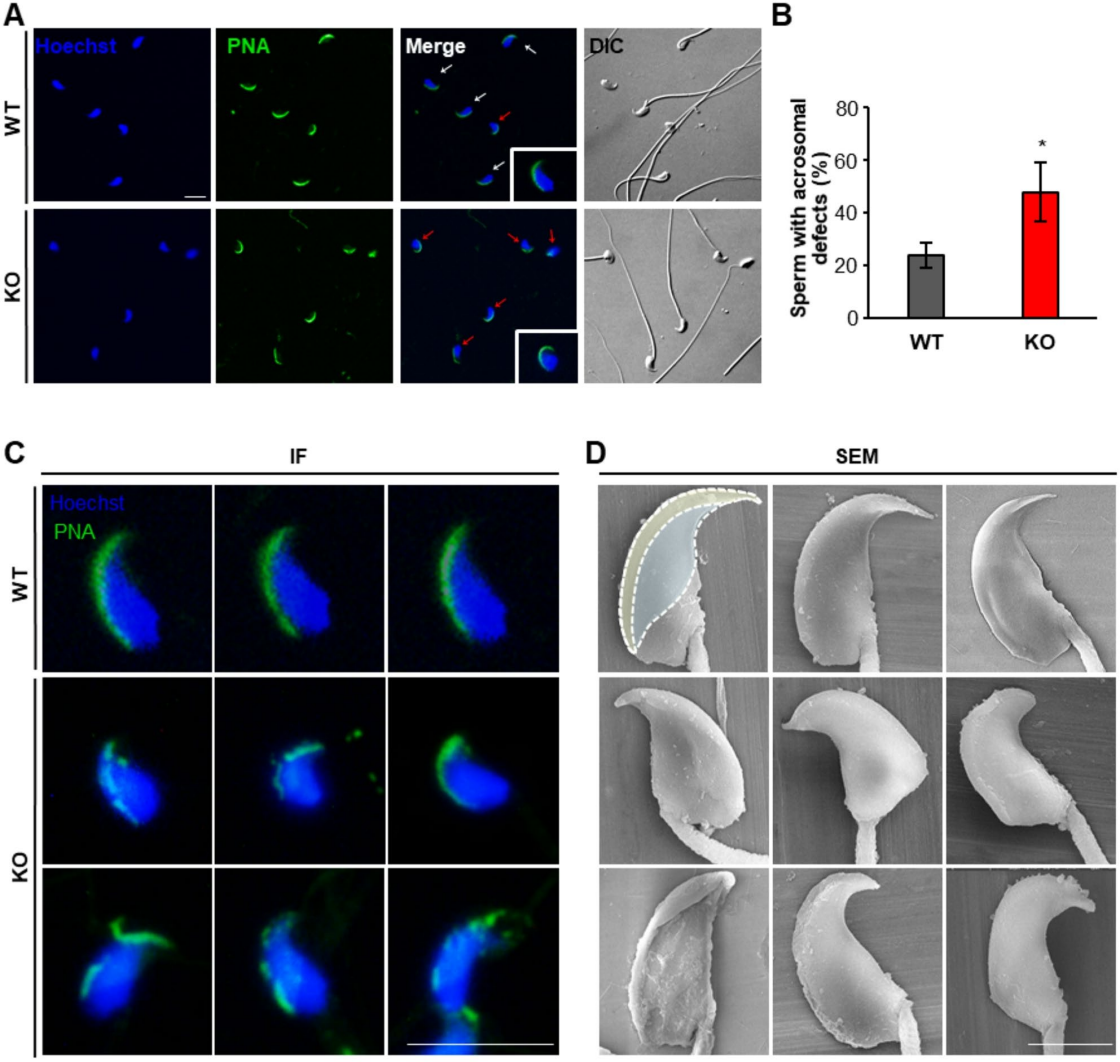
Characterization of acrosome morphology of *Teshl*-KO sperm. Comparison of sperm acrosome morphology by lectin-PNA. **A** Representative epididymal sperm from WT and *Teshl*-KO mice, stained with lectin-PNA and Hoechst. White arrowheads, sperm with normal acrosome; red arrowheads, sperm with abnormal acrosome. Scale bar: 25 μm. **B** Bar graph comparing the proportion of sperm with acrosomal defects between WT and *Teshl*-KO mice, identified by PNA staining. Data are presented means ± SEM (*n* = 3; *P < 0.05, two-tailed Student’s *t* test). **C** Magnified image of a representative lectin-PNA–stained sperm. Scale bar: 25 μm. **D** SEM image showing the head morphology of sperm. Yellow shading, anterior acrosome (AA); blue shading, equatorial segment (ES) region. Scale bar: 3 μm

To further elucidate the cause of the abnormal sperm head morphology in *Teshl-*KO sperm, we examined not only the nucleus but also the acrosome, and closely investigated potential damage to these structures. Defects in sperm morphogenesis can result from nuclear DNA damage associated with defective histone condensation or from impaired acrosomal development. We first performed acrosome staining using lectin-PNA, which allows visualization of overall acrosome shape and localization (Fig. 3A) [23, 24]. A quantitative analysis showed that the proportion of sperm with abnormal acrosome morphology was significantly higher (~ 2.0-fold) in *Teshl*-KO mice (48%) than in WT mice (24%) (Fig. 3B). To further characterize acrosome morphology, we compared magnified lectin-PNA–stained images with SEM images (Fig. 3C, D). This analysis demonstrated that membrane structures of acrosomes from *Teshl*-KO sperm were disrupted or rugged in appearance compared with WT sperm, particularly in the anterior acrosome (AA) of most abnormal sperm; the same also applied to equatorial (ES) segment regions, although to a lesser extent [5].

Toluidine blue staining, performed as previously described [25, 26] to assess nuclear damage, showed no significant difference in DNA integrity between *Teshl*-KO and WT sperm, consistent with previously conducted DNA stability assay results [19] (Supplementary Data 5 A, B). Although we cannot rule out minor alterations in nuclear integrity (Fig. 2), these observations suggest that severe DNA fragmentation or defects in histone condensation are less likely to distinguish *Teshl*-KO sperm from WT sperm. They are also consistent with results showing that embryos fertilized by *Teshl*-KO sperm developed normally and that changes in nuclear shape in the KO sperm were somewhat mild (Figs. 1C and D and 2D). Collectively, our findings suggest that abnormal sperm head morphology and functional abnormalities of sperm in *Teshl*-KO mice are largely attributable to defects in acrosome structure.

### Acrosome morphogenesis during spermiogenesis is abnormal in *Teshl*-KO mice

**Fig. 4:**
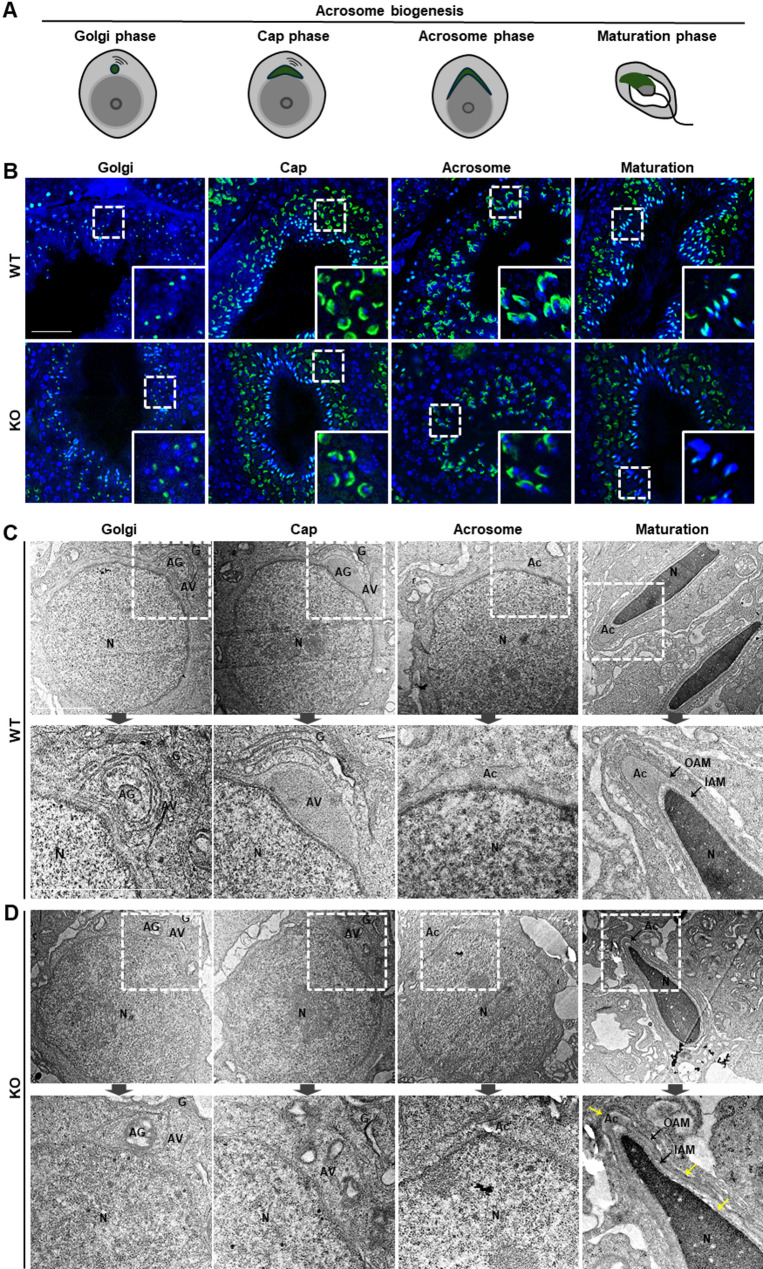
Morphological assessment of the acrosome biogenesis during spermiogenesis in *Teshl*-KO mice. **A** Schematic representation of the phases of acrosome biogenesis: Golgi phase, cap phase, acrosome phase, and maturation phase. **B** Lectin-PNA–stained cross-sections of testes from WT and *Teshl*-KO mice at the four phases of acrosome biogenesis. Dotted boxes show a representative image and solid boxes show a magnified image. Scale bar: 50 μm. **C**, **D** Cross-section of testes from WT (**C**) and *Teshl*-KO (**D**) mice at four phases of acrosome formation, analyzed by TEM. White boxes in the top figures show the developing acrosome, and bottom figures show a magnified image of the box. Yellow arrows indicate abnormal hook shapes and acrosome membrane. *G* Golgi apparatus, *AG* acrosomal granule, *AV* acrosomal vesicle, *N* nucleus, *Ac* acrosome, *OAM* outer acrosomal membrane, *IAM* inner acrosomal membrane, Scale bar: 2 μm

**Fig. 5:**
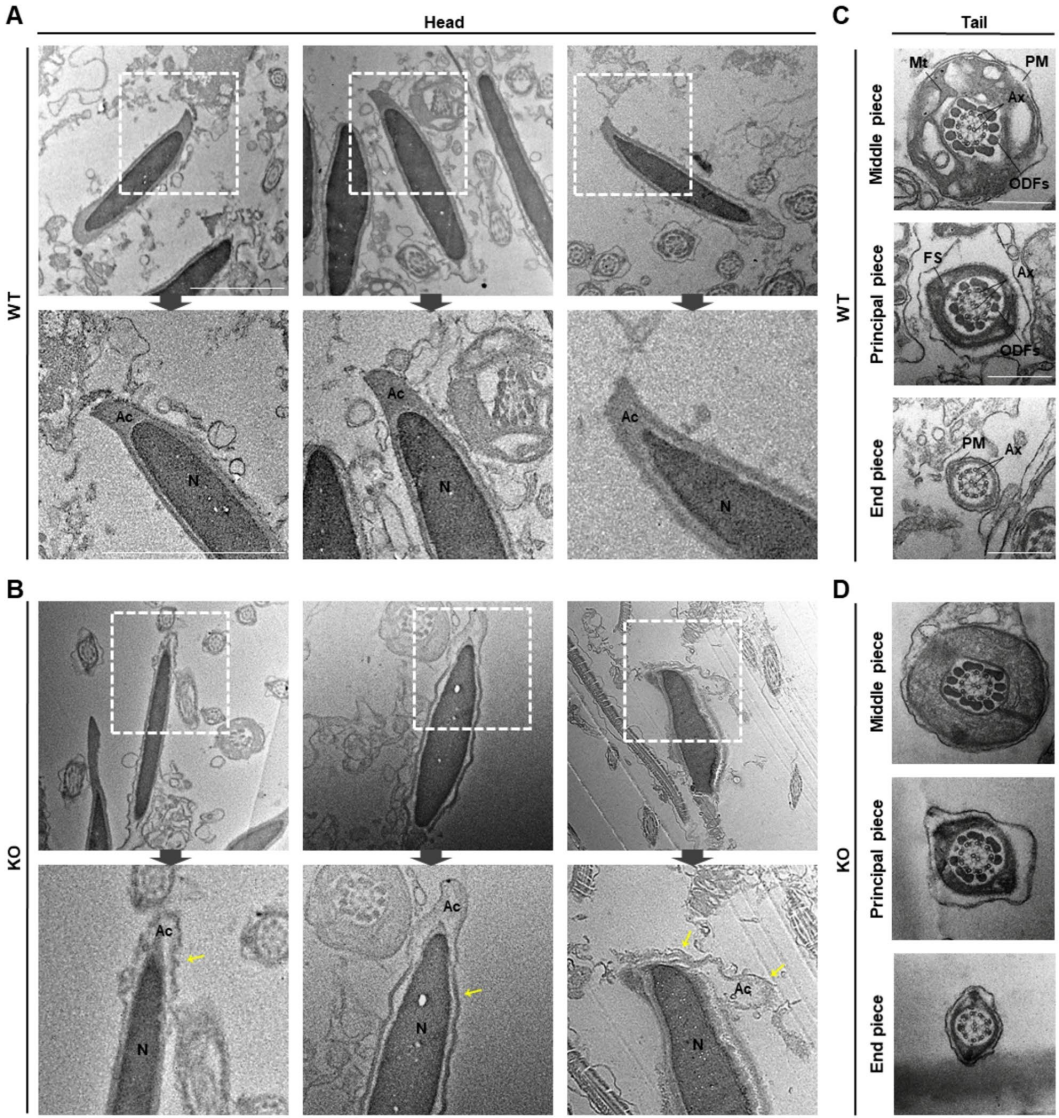
Ultrastructures of *Teshl*-KO sperm. **A**, **B** Cross-section of epididymal sperm from WT (**A**) and *Teshl*-KO (**B**) mice. White boxes show acrosome regions that exhibit morphological differences between WT and *Teshl-*KO mice. Yellow arrows indicate abnormal hook shapes and acrosome membrane. N, nucleus; Ac, acrosome; Scale bar: 1 μm. **C**, **D** Cross-sections of the three structures – middle piece, principal piece and end piece – of the epididymal sperm tail from WT (**C**) and *Teshl*-KO (**D**) mice. *ODFs* outer dense fibers, *Mt* mitochondria, *PM* plasma membrane, *Ax* axoneme, *FS* fibrous sheath, Scale bar: 0.5 μm

To determine whether acrosomal deformities arise during spermiogenesis, we analyzed testis sections, monitoring acrosome structures throughout the four stages of acrosome biogenesis (Golgi, cap, acrosome, and maturation phases) (Fig. 4A) [27]. To this end, we performed lectin-PNA staining to visualize the pattern of acrosome formation, and TEM to analyze ultrastructural features of acrosome development. Lectin-PNA staining showed no differences between WT and *Teshl*-KO spermatids in the Golgi phase, in which the acrosome appears as granules attached to the nuclear surface, or the cap phase, in which these granules spread along the nuclear surface to form a cap-like structure. However, in acrosome and maturation phases, which are the major stages of acrosome condensation, *Teshl*-KO spermatids exhibited weaker fluorescence intensity together with abnormally narrow localization of the acrosome at the nuclear surface compared with WT sperm (Fig. 4B). Consistent with this, the TEM analysis showed no apparent differences in the structures of granules or vesicle membranes in Golgi and cap phases between WT and *Teshl*-KO spermatids, but revealed irregularly shaped membrane structures between the nucleus and acrosome in *Teshl*-KO spermatids during the acrosome phase. In the maturation phase, the acrosomal hook protrusion in *Teshl*-KO spermatids appeared smaller and thinner, and the outer acrosomal membranes (OAMs) and inner acrosomal membranes (IAMs) both appeared irregular or rugged compared with the smooth appearance of WT sperm (Fig. 4C, D). These findings were further confirmed by TEM analysis of mature sperm (Fig. 5A, B). Specifically, a cross-sectional analysis of sperm obtained from the epididymis revealed significant abnormalities in acrosome structure in *Teshl*-KO sperm, including smaller and damaged acrosomal hook structures; apparently damaged OAM and IAM structures; and an atypical acrosomal distribution, with an increase in acrosomes that were not closely attached to the nucleus. However, a cross-sectional analysis of the three structures of the sperm tail (middle piece, principal piece and end piece) revealed no differences between WT and *Teshl-*KO sperm (Fig. 5C, D). Our findings indicate that abnormal acrosome formation during spermiogenesis leads to defective acrosome structures, which ultimately contribute to the aberrant sperm head morphology observed in *Teshl*-KO mice.

### Defective acrosome morphology impairs sperm functions required for fertilization

**Fig. 6:**
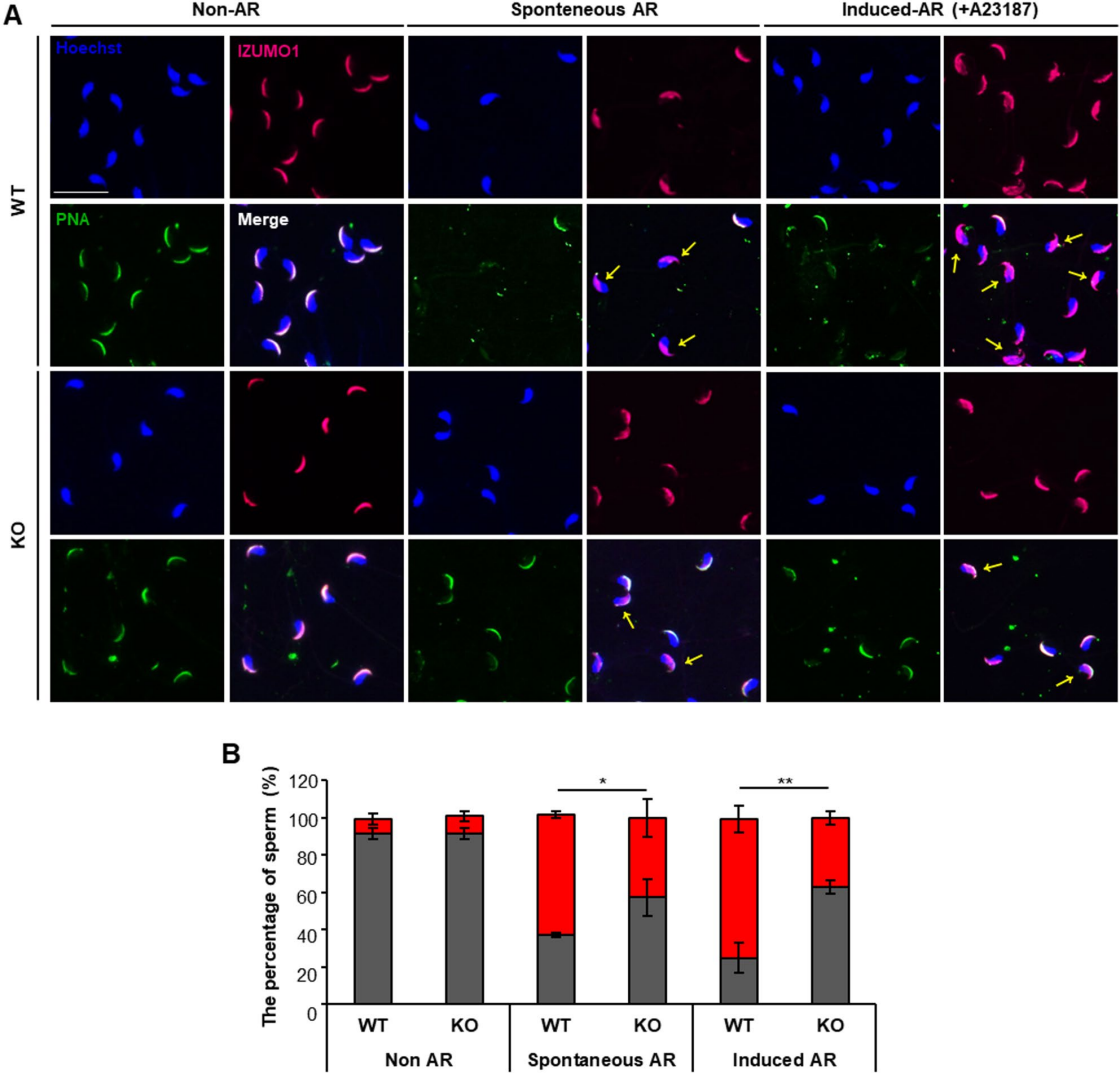
Analysis of the acrosome reaction in *Teshl*-KO sperm. **A** Staining shows the AR of sperm. Hoechst was used for nuclear staining, and PNA and IZUMO1 were used for acrosome staining. Non-AR indicates non-acrosome reaction (i.e., without incubation). For spontaneous AR, sperm were incubated with YTH media for 4 h. In the case of induced-AR, sperm were incubated with A23187 for 10 min. Yellow arrowheads indicate reacted sperm. Scale bar: 20 μm. **B **Percentage of sperm with AR (red bars) in WT and *Teshl*-KO mice. Data are presented as means ± SEM (*n* = 3; *P < 0.05, **P < 0.01, two-tailed Student’s *t* test)

**Fig. 7:**
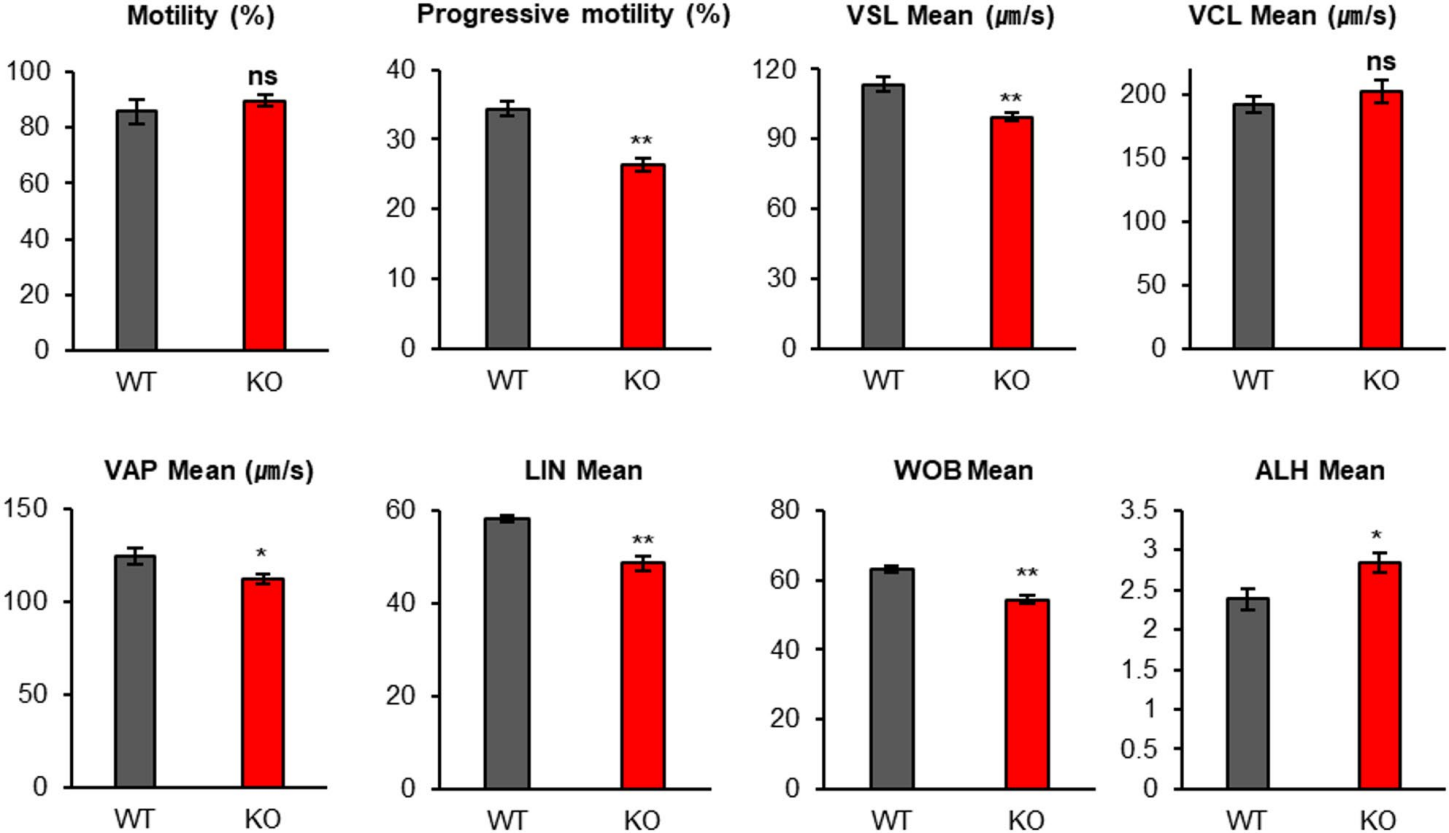
Motility of *Teshl*-KO sperm. Sperm motility was analyzed based on 3-s videos of sperm for each mouse, recorded using openCASA. The following parameters of motility were measured: percentage of total motility, progressive motility, straight-line velocity (VSL), curvilinear velocity (VCL), average-path velocity (VAP), linearity (LIN; [VSL/VCL]), wobble coefficient (WOB; [VAP/VCL]), and amplitude of lateral head displacement (ALH). Data are presented as means ± SEM (*n* = 3; *P < 0.05, **P < 0.01, two-tailed Student’s *t* test)

The acrosome reaction, a key function of the acrosome, is an exocytotic event that occurs within the acrosome. Previous studies have shown that abnormalities in the acrosome of the sperm head impair the acrosome reaction [28, 29]. To determine whether the acrosome reaction is abnormal in *Teshl*-KO sperm in which acrosome defects were observed, we analyzed the capacity of sperm to undergo the acrosome reaction (Fig. 6A) by monitoring IZUMO1 (izumo sperm-egg fusion 1) [30], an acrosomal protein whose expression pattern changes depending on acrosome-reaction status, to distinguish between acrosome-reacted (AR) and acrosome-intact (AI) sperm. Immediately after sperm were collected from the epididymis (i.e., prior to culture in vitro), there was no significant difference in the proportion of AR sperm between WT and *Teshl*-KO mice (Fig. 6B). However, after a 4-hour incubation in TYH medium, the proportion of AI sperm was significantly increased in *Teshl*-KO mice compared with WT mice, both under conditions of spontaneous acrosome reactions (WT, 36%; KO, 57%) and acrosome reactions induced by treatment with the calcium ionophore, A23187 (WT, 25%; KO, 63%) (Fig. 6B) [31]. These results indicate that the ability to undergo the acrosome reaction is impaired in sperm from *Teshl*-KO mice compared with sperm from WT mice. Additionally, we performed a sperm motility analysis in which epididymal sperm were capacitated in TYH medium and various motility parameters were assessed by light microscopy with the assistance of openCASA [32], an open-source, computer-assisted semen analysis program (Fig. 7) [33]. Although the proportion of motile sperm did not differ significantly between *Teshl*-KO and WT mice, *Teshl*-KO sperm exhibited reduced progressive motility reflecting impaired linear and forward movement. Consistent with this, other key parameters critical for normal sperm migration, including straight-line velocity (VSL), average-path velocity (VAP), linearity (LIN), wobble coefficient (WOB) and amplitude of lateral head displacement (ALH), were also decreased in *Teshl*-KO sperm, indicating that a *Teshl* deficiency impairs sperm motility, which is generally required for successful fertilization. Taken together, our findings indicate that *Teshl*-KO mice display impaired sperm functions, with specific defects in the acrosome reaction and motility. This suggests that acrosome-associated morphological abnormalities contribute to sperm dysfunction and, ultimately, reduced fertilization efficiency.

### *Teshl* is involved in the regulation of multiple acrosomal proteins

**Fig. 8:**
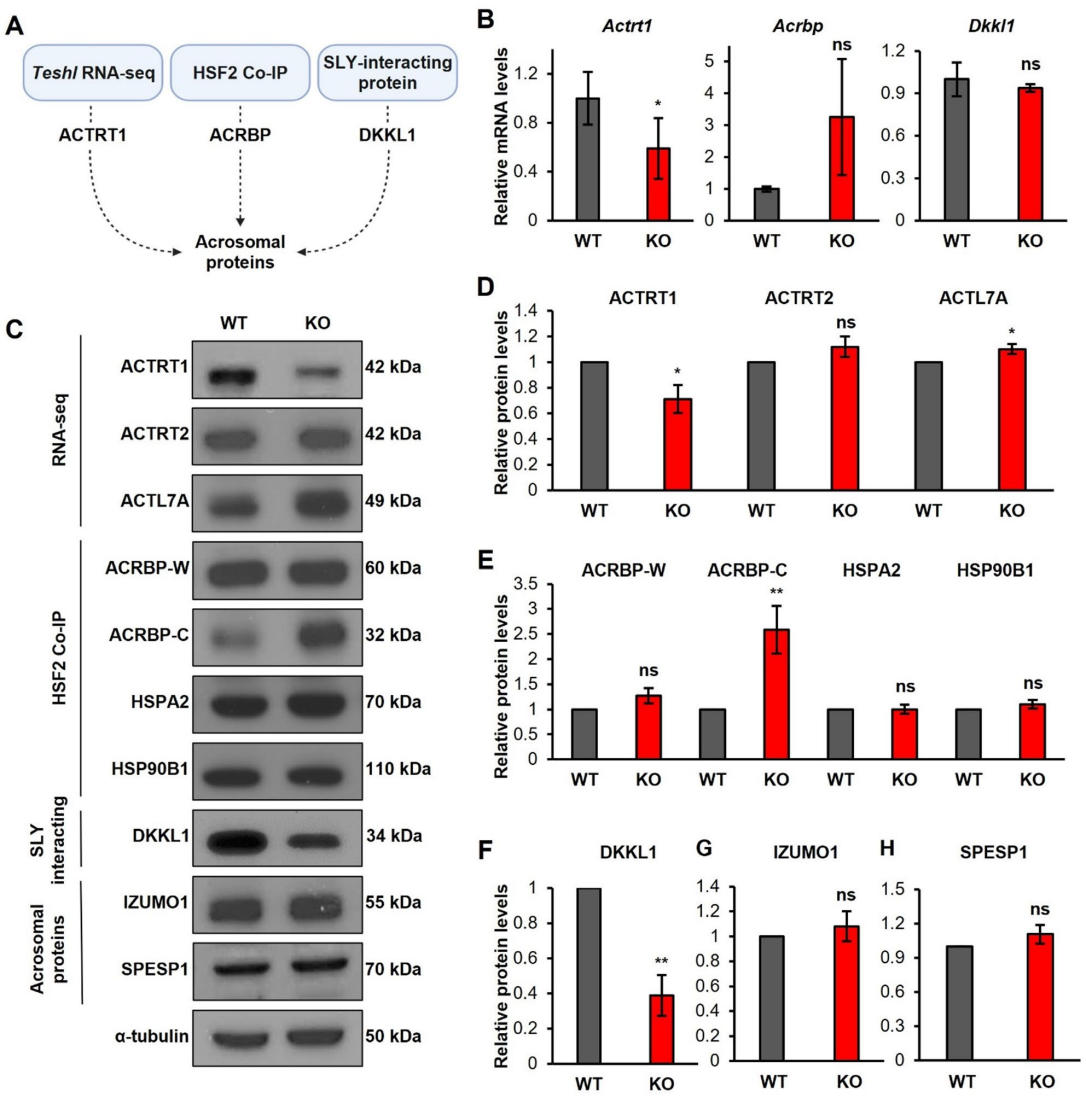
Expression analysis of *Teshl*-related acrosomal proteins in *Teshl*-KO mice. **A** Schematic diagram showing candidate *Teshl*-associated acrosomal proteins derived from three data sets: *Teshl* RNA-seq, HSF2 co-immunoprecipitation, and SLY-interacting proteins. **B** qRT-PCR analysis of *Teshl-*associated acrosomal genes. *Gapdh* mRNA was used as a control. Data are presented as means ± SD (*n* = 3; *P < 0.05, two-tailed Student’s *t* test). **C–H** Immunoblot analysis of acrosomal proteins identified from multiple datasets: **C** Representative Western blot data; **D** *Teshl* RNA-seq; **E** HSF2 co-immunoprecipitation; **F** DKKL1, a SLY-interacting acrosomal protein; **G** IZUMO1, representative acrosomal protein; and **H** SPESP1, another representative acrosomal protein. Protein levels were assessed by Western blot analysis and normalized to α-tubulin. Data are presented as means ± SEM (*n* = 3; *P < 0.05, **P < 0.01, two-tailed Student’s *t* test)

To uncover the molecular basis underlying the sperm head damage observed in *Teshl*-KO mice, we investigated candidate molecules selected from information available in three different datasets: *Teshl* RNA-sequencing (RNA-seq), HSF2 co-immunoprecipitation (co-IP), and SLY-interacting proteins (Fig. 8A). First, for the *Teshl* RNA-seq dataset, we analyzed previously generated RNA-seq results from WT and *Teshl*-KO testes [19]. Among 82 differentially expressed genes (DEGs; fold change > 1.5, *P* < 0.05), we identified *Actrt1* as showing altered expression. The protein encoded by this gene, ACTRT1, is a sperm head structure-associated PT protein that plays a crucial role in anchoring the acrosome to the nuclear envelope. Next, through analysis of the HSF2 co-IP dataset in testes, we identified ACRBP as a protein associated with HSF2, a *Teshl*-interacting protein that functions through interactions with transcription factors and various proteins [34]. Finally, an analysis of proteins interacting with SLY, a major downstream binding target of *Teshl*, identified the acrosomal protein DKKL1 [35]. An analysis of mRNA levels of the three candidates showed that *Actrt1* transcript levels were markedly reduced in *Teshl*-KO testes, whereas the levels of *Acrbp* and *Dkkl1* mRNAs showed no significant differences between the two genotypes (Fig. 8B).

We next analyzed expression levels of candidate proteins as well as other proteins reported to interact with the candidates or possibly show a relationship with them (Fig. 8C). Expression levels of ACTRT1 protein were significantly decreased in *Teshl*-KO testes; two other testes ACTRT1-interacting proteins – the related ACTRT2 and ACTL7A – were unchanged or slightly increased, respectively (Fig. 8D). *Actrt1*, and possibly *Actl7a*, can be considered targets of *Teshl*-mediated gene regulation. We then examined ACRBP and two interacting heat-shock proteins, HSPA2 and HSP90B1. Of these, only ACRBP showed a significant increase in expression. Notably, this increase was observed in the form of the post-translationally modified variant, ACRBP-C, suggesting that a *Teshl* deficiency impairs normal levels of ACRBP protein in mice (Fig. 8E). ACRBP-C is generated through post-translational processing of the full-length ACRBP-W protein. While the total abundance of ACRBP is known to remain relatively constant during spermatogenesis, the relative ratios of its isoforms shift as ACRBP-W is proteolytically cleaved into ACRBP-C. Notably, our data demonstrate that *Teshl* deficiency selectively alters the levels of the ACRBP-C variant without affecting the primary ACRBP-W form. These findings suggest that *Teshl* may play a selective role in regulating the stability or processing of this specific variant, a possibility whose underlying mechanism warrants further investigation. Expression levels of DKKL1 protein were significantly reduced in *Teshl*-KO testes, whereas the levels of IZUMO1 and SPESP1, which are representative acrosomal proteins but are not considered to be regulated by *Teshl* [36], remained unchanged (Fig. 8F–H). Immunostaining analyses also revealed that DKKL1 exhibited an abnormal, weak and fuzzy distribution pattern in *Teshl*-KO sperm heads (Supplementary Data 6 A, B). These data suggest that the acrosome protein DKKL1 is selectively regulated by *Teshl.* Proteins selected from among candidate acrosomal proteins predicted to be linked to *Teshl* regulation were indeed found to exhibit altered expression at gene and/or protein levels in *Teshl*-KO testes. These findings suggest that *Teshl* regulates specific acrosomal proteins, either directly or indirectly, through multiple pathways, thereby contributing to normal sperm head formation – particularly acrosome formation – and thus sperm function.

## Discussion

In this study, we used a series of in-depth phenotypic analyses employing *Teshl*-KO mice to investigate the function of *Teshl* during spermatogenesis. A *Teshl* deficiency was previously shown to result in three abnormal phenotypes: an increased proportion of sperm with abnormal heads, a reduced number of offspring, and a skewed sex ratio among offspring [19]. Of these, the most profound difference in *Teshl*-KO mice compared to WT mice was the abnormal sperm head phenotype, which likely represents an epistatic phenotype that might influence the other two reproductive outcomes [37–39]. This interpretation is consistent with previous studies reporting that sperm head morphology is a critical determinant of fertilization efficiency [40, 41]. Based on these observations, we focused on the functional and mechanistic roles of *Teshl* in spermatogenesis in relation to these abnormal phenotypes, with a particular emphasis on the abnormal sperm head morphology observed in *Teshl*-KO mice.

Our morphological analyses demonstrated that deletion of *Teshl* led to abnormal sperm head morphology secondary to acrosomal malformation. Although a software-based nuclear morphology analysis of nucleus-stained samples revealed abnormalities in head shape, the nuclear shape abnormalities that were detected were very mild and were limited in their ability to clearly demonstrate a causal relationship between these abnormalities and impaired fertilization. Condensation of the acrosome and nucleus occur simultaneously during late spermiogenesis and influence each other structurally. However, the precise interrelationships between these processes remain unresolved [10, 42].The stability of the acrosome is primarily determined by its position and structural integrity, reflecting its formation through the cooperative participation of multiple proteins [43], whereas stability of the nucleus relies on DNA compaction through appropriate chromatin condensation. Our analyses revealed no evident defects in nuclear condensation in *Teshl*-KO sperm, but did show consistent and distinct abnormalities in acrosomal structures. These acrosome defects in *Teshl*-KO spermatids and mature sperm were observed in detail by TEM analysis, which showed small and poorly distributed acrosomes and abnormalities in the acrosomal membrane. These findings suggest that *Teshl* is essential for normal acrosome development and thus establishment of the structural integrity of the sperm head.

We found that acrosomal defects in *Teshl*-KO sperm led to acrosomal dysfunction in the form of an impaired acrosome reaction, suggesting that this functional consequence of acrosomal abnormalities may primarily underlie fertility problems observed in *Teshl*-KO mice. The acrosome reaction is a key and essential function of the acrosome, and the associated extracellular secretion supports the activity of specific acrosomal proteins that allow sperm to penetrate the zona pellucida of the egg and fuse with the egg cell membrane, which are essential for fertilization to occur [44–46]. While the possibility that *Teshl* might directly regulate the sperm acrosome reaction cannot be ruled out, it is more likely that it acts as an upstream regulator of molecules involved in acrosome formation – a necessary prerequisite for a normal acrosome reaction. Interestingly, we also found that *Teshl*-KO sperm exhibited reduced motility. Sperm motility can be affected by defects in either the tail or head [41, 47]. Structural abnormalities in the tail were absent in *Teshl*-KO sperm, suggesting that abnormal head morphology rather than tail morphology is the likely cause. The relationship between motility and sperm head morphology is not fully understood; however, previous studies in mice have suggested that a hook-shaped sperm head facilitates migration through the female reproductive tract [12, 48]. Although we did not directly examine sperm migration within the tract, *Teshl*-KO sperm displayed alterations in parameters related to directionality, despite no significant difference in the percentage of motile sperm. Perhaps the head deformation of *Teshl-*KO sperm alters their orientation to fluid flow (rheotaxis) observed during sperm motility. Alternatively, it is possible that *Teshl* regulates proteins that are not involved in sperm tail structure but are functionally involved in sperm motility. Further research is needed to address this issue.

**Fig. 9:**
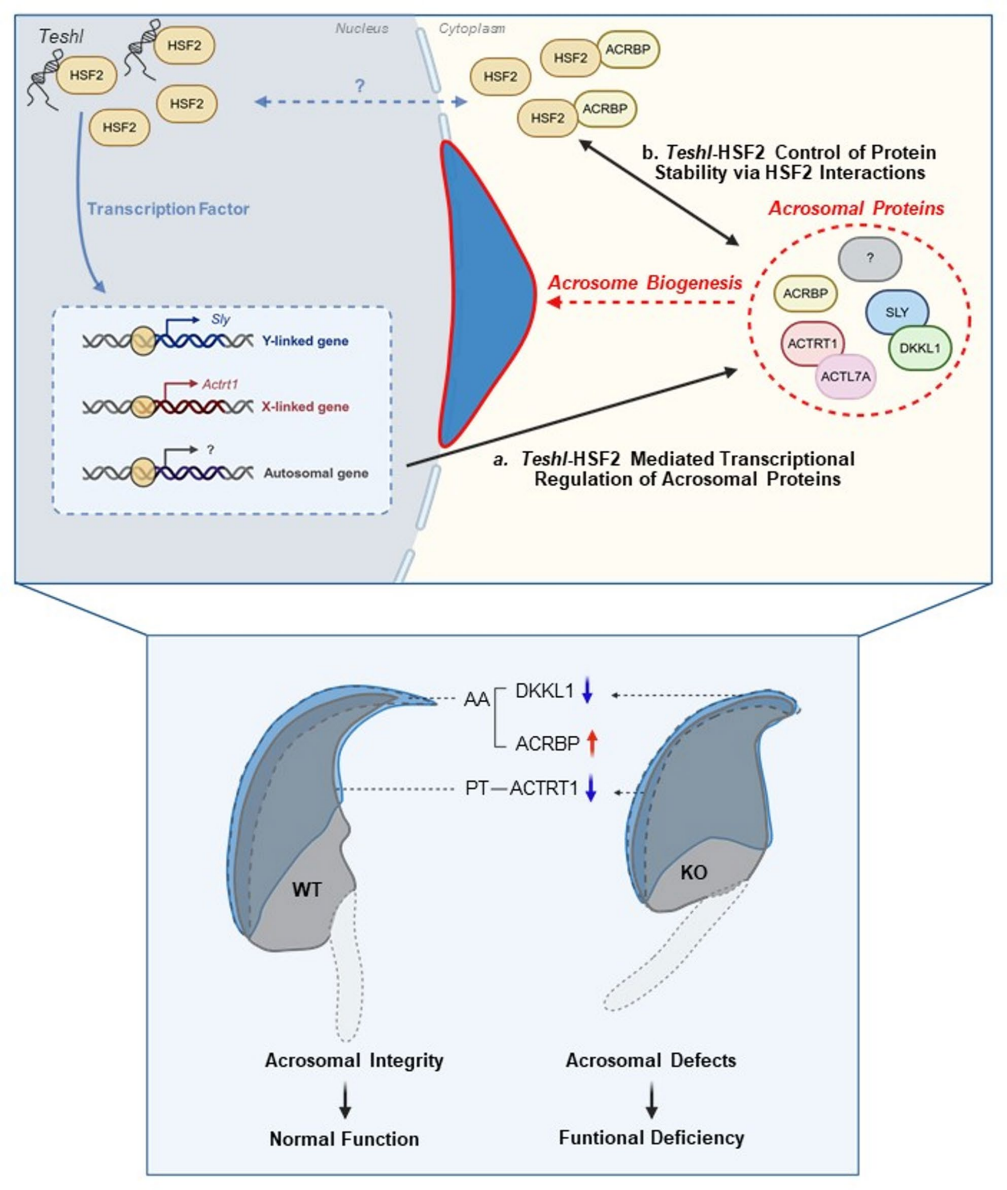
Schematic diagram depicting two potential mechanisms by which *Teshl*-regulates acrosome biogenesis.** a** *Teshl*-HSF2–mediated transcriptional regulation of acrosomal proteins: *Teshl* is known to localize to the nucleus, where a portion interacts with the transcription factor HSF2 during spermiogenesis (Ref. 19). The *Teshl*-HSF2 complex regulates the transcription of *Sly* (Ref. 19, 50) and may also regulate that of the acrosome gene *Actrt1*, based on previous RNA-seq analyses (Ref. 19). SLY has been shown to interact with the acrosome protein DKKL1 (Ref 35). Thus, taken together, the described findings suggest that this complex directly regulates *Actrt1* transcription and indirectly influences DKKL1 through transcriptional regulation followed by protein interaction with SLY. **b** *Teshl*-HSF2–mediated control of protein stability via HSF2 interactions: Although the mechanism governing HSF2 nuclear–cytoplasmic localization remains unclear, it is hypothesized that *Teshl* modulates the abundance of cytoplasmic HSF2 (Ref. 34) via its interaction with nuclear HSF2. This regulation may influence downstream effects of *Teshl*-free HSF2, including its association with effectors such as ACRBP, thereby maintaining the normal level or integrity of ACRBP. Through these two mechanisms, *Teshl* contributes to acrosome biogenesis, which is essential for sperm function and fertilization. Acrosomes and AA (anterior acrosome) are indicated by the blue regions and dotted lines, respectively. PT (perinuclear theca) is represented as a layer surrounding the nucleus. Red arrow, up- regulation; blue arrow, down-regulation. *Figure created with BioRender.com*

Importantly, our study utilized a rigorous bottom-up approach, leveraging all available databases – *Teshl* RNA-seq, HSF2 co-IP, and SLY-interacting proteins – to identify candidate acrosomal proteins regulated by or related to *Teshl* and provide insight into the molecular pathways by which *Teshl* contributes to acrosome biogenesis. From the *Teshl* testis RNA-seq dataset, we identified *Actrt1* as the only acrosome-related gene whose expression was altered in *Teshl*-KO testes. ACTRT1 is an X-linked PT protein that acts in cooperation with other PT, acrosomal, and nuclear membrane proteins to play an essential role in maintaining sperm head structure and function. Consistent with this, *Actrt1*-KO mice display sperm head abnormalities and subfertility, phenotypes similar to those observed in *Teshl*-KO mice [6, 49]. These findings strongly suggest that *Teshl* contributes to sperm head integrity through regulation of ACTRT1. Whether the *Teshl*–HSF2 complex directly regulates *Actrt1* transcription—specifically through binding to the enhancer or promoter regions of the *Actrt1* gene—remains to be determined in future studies. HSF2 co-IP analyses identified ACRBP as another *Teshl*-related protein. ACRBP interacts with HSF2, which is the only protein identified to date that directly binds *Teshl* [50]. In addition to its role as a transcription factor, HSF2 also functions at the nuclear periphery and in the cytoplasm through interactions with other proteins. These non-canonical activities prompted us to examine whether HSF2 might have *Teshl*-associated roles at the protein level beyond its role as a transcription factor – an examination that identified ACRBP among HSF2-interacting proteins. ACRBP is essential for acrosome biogenesis and regulates acrosin activation during the acrosome reaction [51, 52]. The observed increase in the expression of a variant form of ACRBP in *Teshl*-KO testes suggest that *Teshl*-HSF2 influences ACRBP post-translational modification and consequent protein stability, thereby affecting acrosomal integrity. Note that the *Teshl-*HSF2 association was demonstrated in our previous study [19]. Finally, our interrogation of the SLY-interacting proteins dataset identified the acrosomal protein DKKL1. *Sly* is a representative Y-linked multicopy gene whose expression is regulated by *Teshl* [53, 54]. *Sly*-KO mice exhibit phenotypes similar to those of *Teshl*-KO mice, including abnormal head appearance, subfertility and distorted sex ratio, together with aberrant DKKL1 expression patterns [55]. Consistent with this, we found that DKKL1 protein levels were reduced and DKKL1 expression patterns were abnormal in *Teshl*-KO mice [35, 56]. Although DKKL1 apparently has no strong effect on sperm head morphology, it is reported to be involved in sperm penetration during fertilization [57–59]. It is possible that altered expression of the identified proteins – ACTRT1, ACRBP and DKKL1 – in *Teshl-*KO cells is not the cause of the acrosomal structural abnormality, but rather the result of abnormal acrosomes caused by other factors. However, this is highly unlikely, as all of these factors are specifically associated with *Teshl*. Taken together, our results suggest that *Teshl*, together with HSF2, directly or indirectly regulates multiple acrosomal proteins through two mechanisms: transcription (*Teshl*-HSF2-*Actrt1*/ACTRT1 and *Teshl*-HSF2-*Sly*/SLY-DKKL1) and protein-protein interaction (*Teshl*-HSF2-ACRBP) (Fig. 9). This indicates that *Teshl* acts not only as a transcriptional regulator but also contributes to acrosomal integrity through broader molecular interactions. It should be noted that ACTRT1, ACRBP and DKKL1 are unlikely to be the only factors sufficient to fully account for the causal relationship between *Teshl* loss and defective acrosome biogenesis. Rather, these proteins represent key candidates identified through integrative analyses of multiple datasets and are likely to function within a broader regulatory network governing acrosome formation. Moreover, the precise molecular mechanisms by which these factors act downstream of the *Teshl*–HSF2 axis remain to be defined. Elucidating how each component contributes to acrosome biogenesis will require further mechanistic studies in the future.

In conclusion, our findings demonstrate that *Teshl* serves as a pivotal regulator of acrosomal integrity in sperm, exemplifying the multifaceted ways in which lncRNAs orchestrate male reproductive biology.

## Supplementary Information

Below is the link to the electronic supplementary material.

Supplementary Material 1.

Supplementary Material 2.

## Data Availability

All data generated or analyzed during this study are included in this published article and its supplementary information files.
