# Supplementary figures and images for: Testis-specific lncRNA Teshl regulates acrosome biogenesis to maintain sperm structure and function
Source: Cell Biosci. 2026 Mar 30;16:55. doi: 10.1186/s13578-026-01563-6 (PMC13154684; doi:10.1186/s13578-026-01563-6)

# Figure 1E

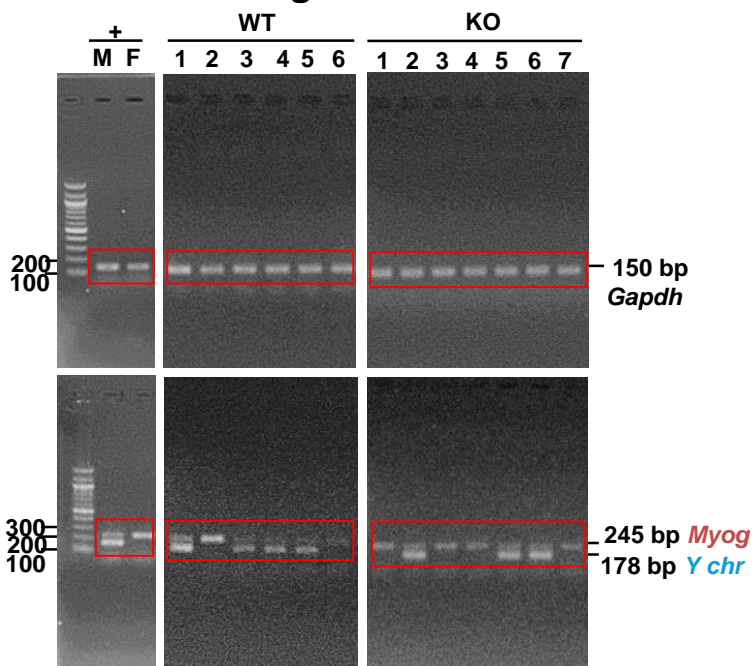

## Supplementary Data 2B

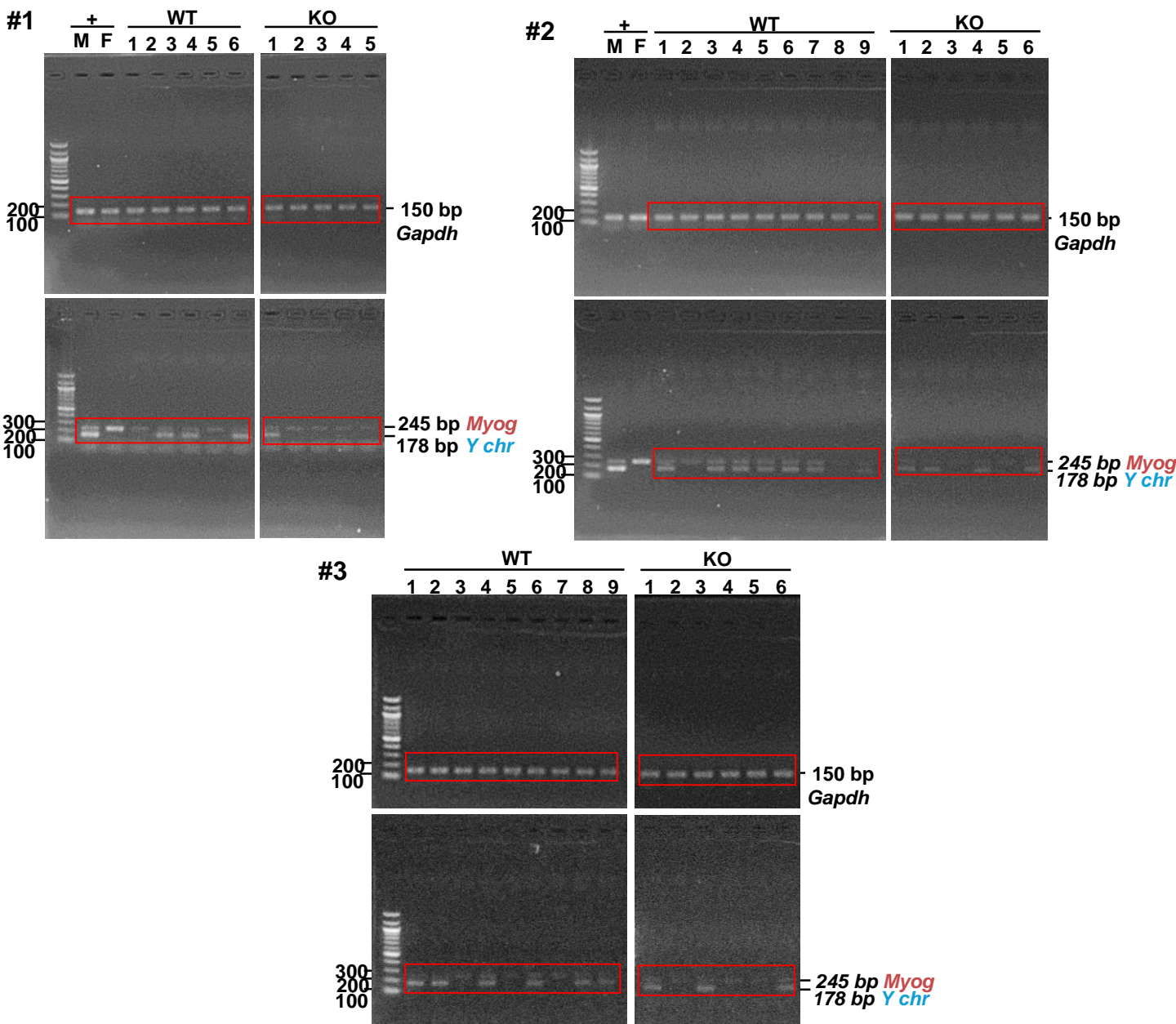

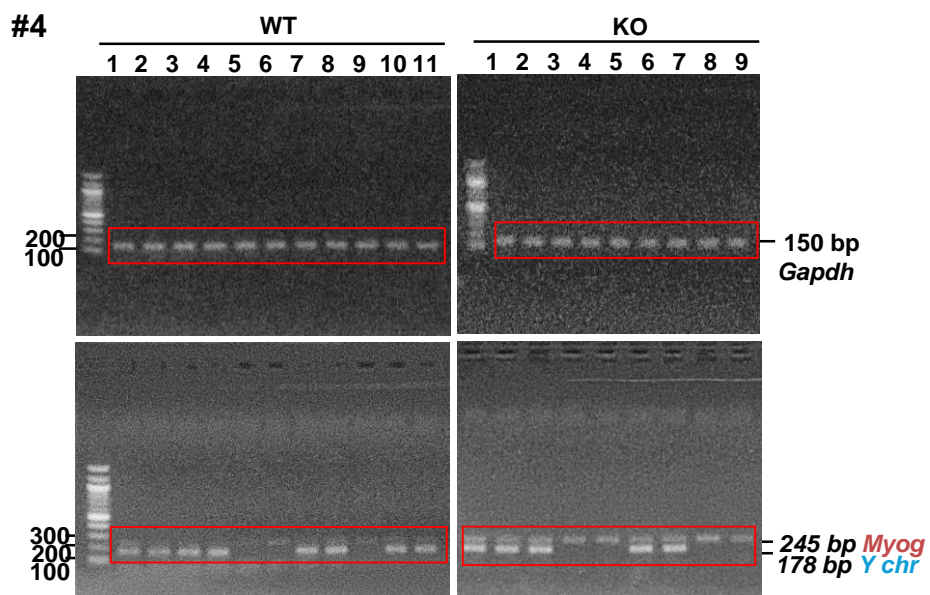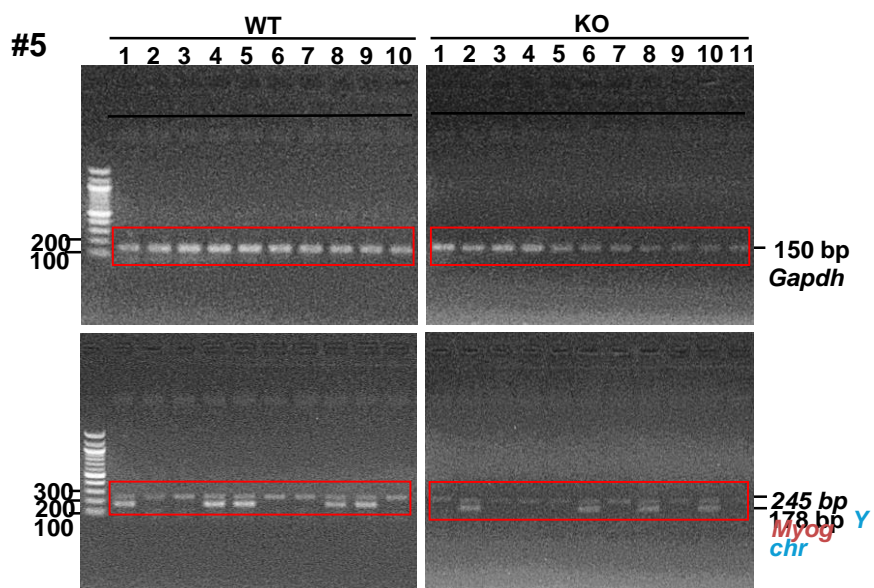

**Figure 8C**

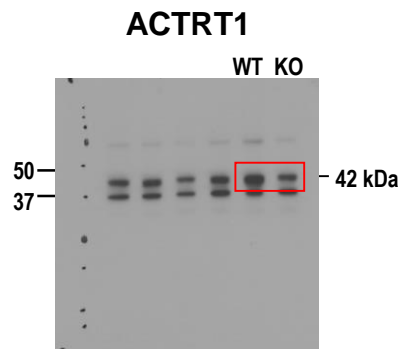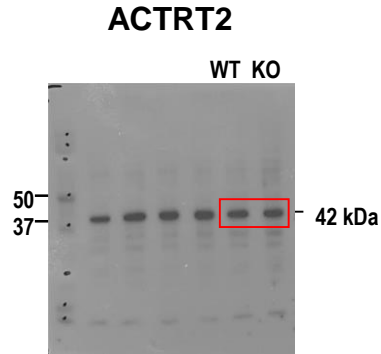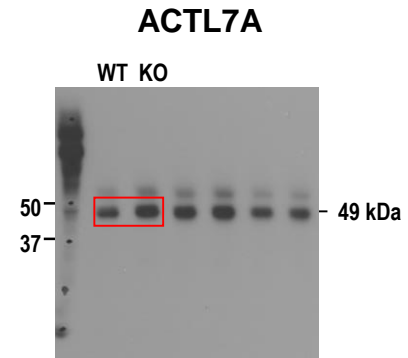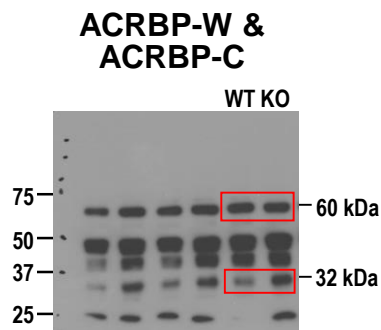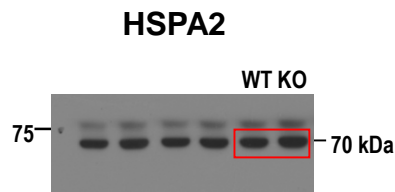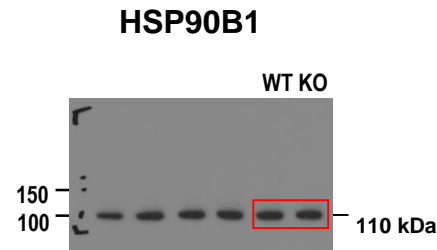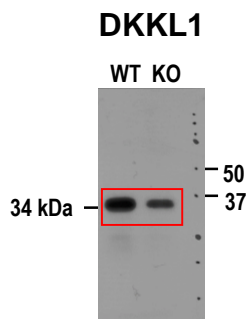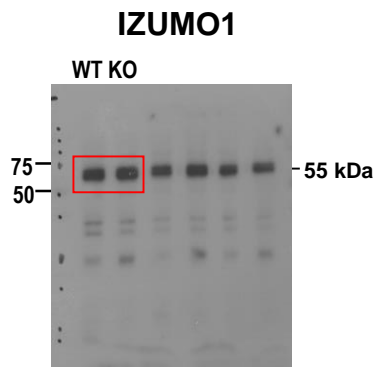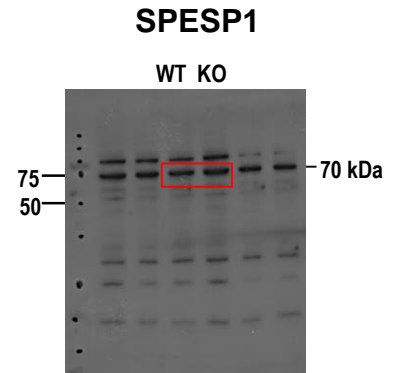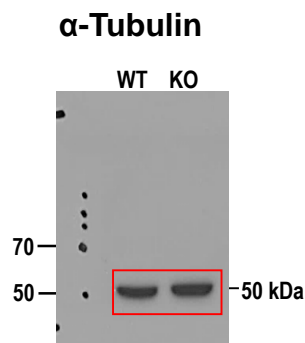

Supplement: Supplementary file 1 — Supplementary Material 1. [file 13578_2026_1563_MOESM1_ESM.pdf]
