# Supplementary material for: Testis-specific lncRNA Teshl regulates acrosome biogenesis to maintain sperm structure and function
Source: Cell Biosci. 2026 Mar 30;16:55. doi: 10.1186/s13578-026-01563-6 (PMC13154684; doi:10.1186/s13578-026-01563-6)
Supplement: Supplementary file 2 — Supplementary Material 2. [file 13578_2026_1563_MOESM2_ESM.pdf]

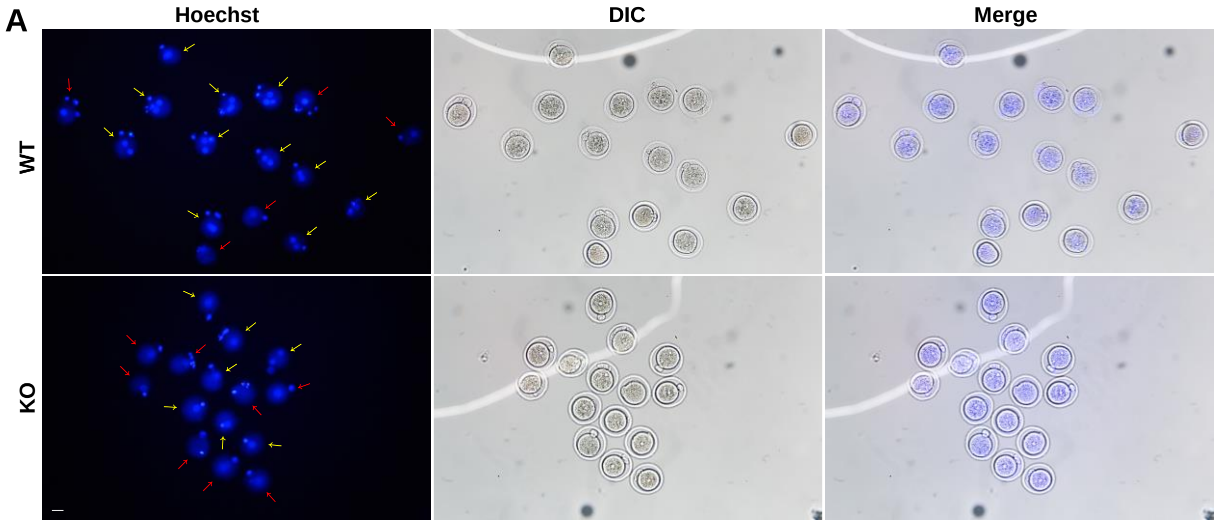

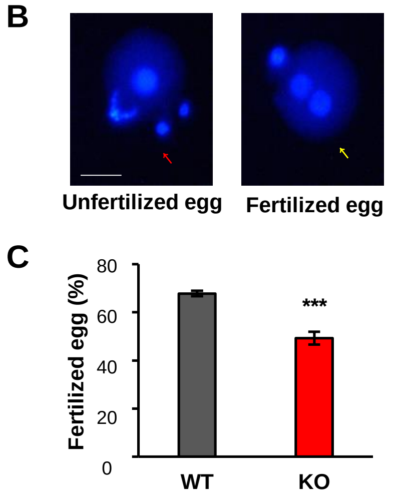

D

	WT	KO
# 1	0.68	0.46
# 2	0.69	0.5
# 3	0.66	0.53
# 4	0.66	0.47
Avg	0.67	0.49
SEM	0.01299	0.027386
Two-tailed Student's *t* test	3.36912E-05	

Supplementary Data 1. Pronuclear formation in zygotes fertilized by *Teshl*-KO sperm.
(A) Immunofluorescence imaging and differential interference contrast (DIC) microscopy of Hoechst-stained embryos after *in vitro* fertilization. Yellow arrow, fertilized egg; red arrow, unfertilized egg. **(B)** Magnified images of representative images of stained unfertilized and fertilized eggs. **(C)** Bar graph showing the percentage of fertilized eggs following exposure to sperm from WT or *Teshl*-KO mice. Data are presented as means ± SEM (n = 4; ***$P < 0.001$, two-tailed Student's *t* test). **(D)** Comparison of fertilization rates of each type of mouse.

A

	2-cells / retrieved eggs		Morula / 2-cells		Blastocyst / 2-cells	
	**WT**	**KO**	**WT**	**KO**	**WT**	**KO**
# 1	0.89	0.70	0.96	0.79	0.29	0.36
# 2	0.75	0.60	0.95	0.93	0.53	0.46
# 3	0.85	0.58	1.00	1.00	0.39	0.43
# 4	0.90	0.68	0.92	0.94	0.58	0.60
# 5	0.79	0.77	1.00	0.85	0.74	0.82
# 6	0.72	0.64	0.67	0.69	0.31	0.44
Avg	0.82	0.66	0.92	0.87	0.47	0.52
SEM	0.06	0.07	0.11	0.10	0.16	0.15
Two-tailed Student's *t* test	0.004		0.49		0.66	

B

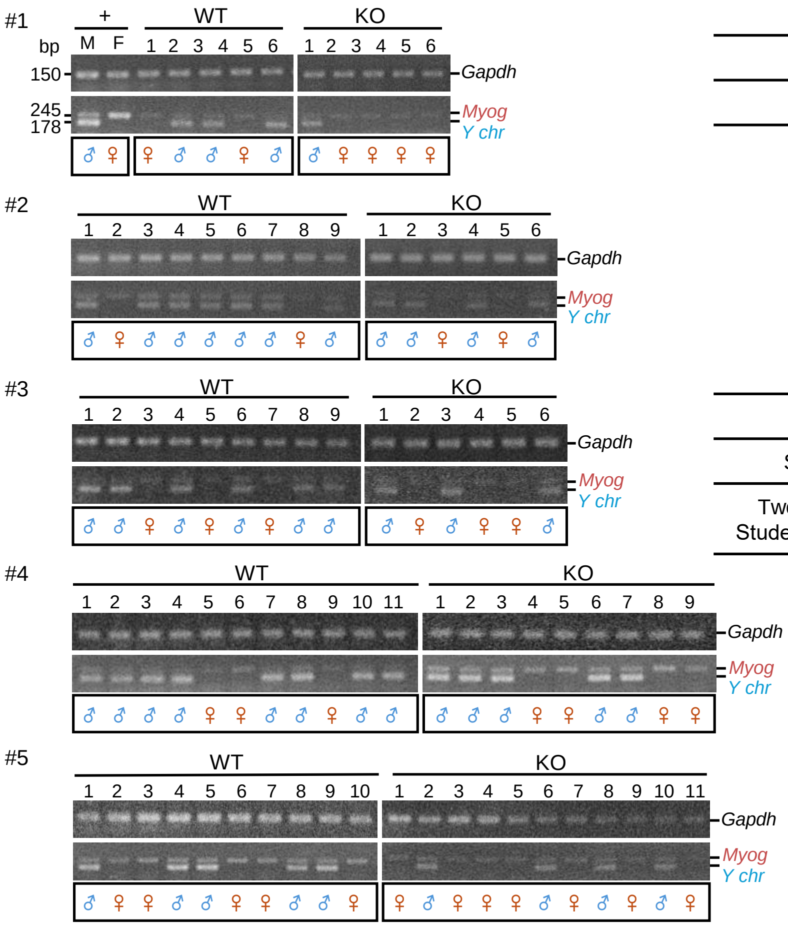

C

	Blastocyst			
	**WT**		**KO**	
	**Male**	**Female**	**Male**	**Female**
# 1	0.60	0.40	0.20	0.80
# 2	0.78	0.22	0.67	0.33
# 3	0.67	0.33	0.50	0.50
# 4	0.73	0.27	0.63	0.38
# 5	0.50	0.50	0.36	0.64
# 6	0.75	0.25	0.43	0.57
Avg	0.67	0.33	0.46	0.54
SEM	0.10	0.10	0.16	0.16
Two-tailed Student's *t* test	0.0315			

Supplementary Data 2. Outcomes of *in vitro* fertilization and blastocyst PCR.
(A) Table summarizing the results of *in vitro* fertilization. **(B)** Sex-determining PCR analysis of blastocysts, obtained using sperm from WT and *Teshl*-KO mice. **(C)** Table summarizing the results of blastocyst PCR. Data are presented as means ± SEM (n = 6; **P* > 0.05, ***P* > 0.01, two-tailed Student's *t* test).

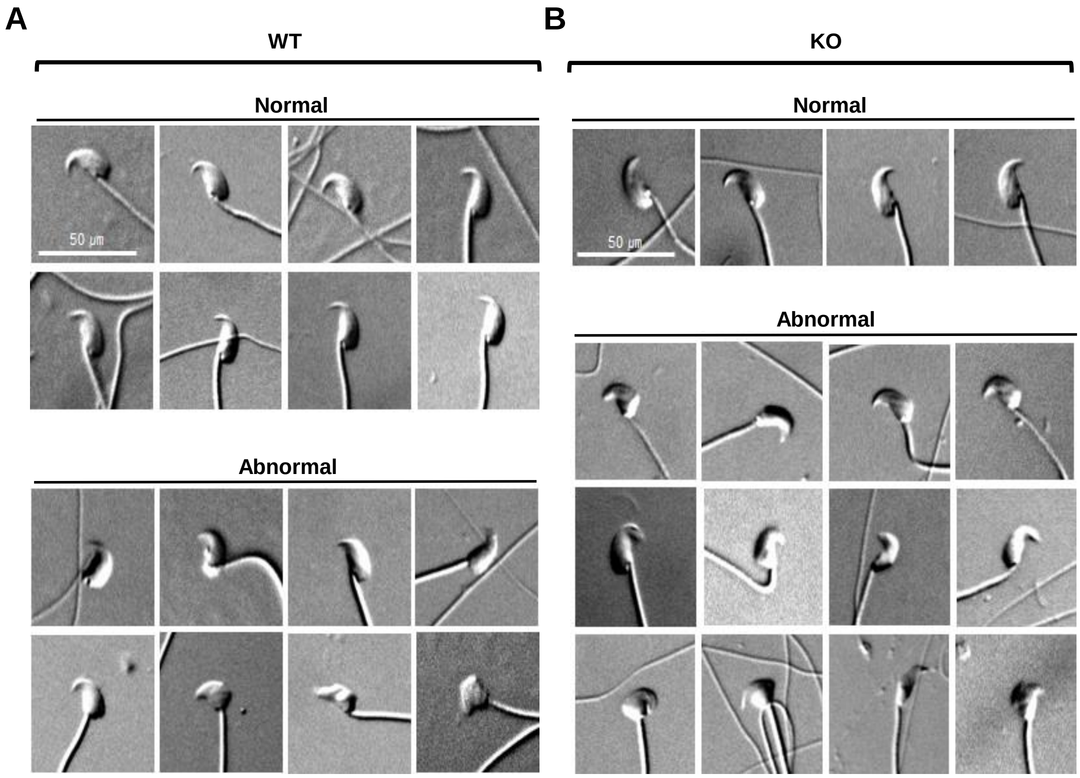

Supplementary Data 3. Bright-field images of sperm from Wild-type and *Teshl*-KO mice.
Bright-field microscopy images showing examples of morphologically normal and various abnormal sperm heads in WT mice **(A)** and *Teshl*-KO mice **(B)**. Scale bars: 50 μm.

	WT	***Teshl*-KO**
Area mean (square pixels)	2528.33	2849.41
Area 95% CI (square pixels)	2528.33 ± 38.72	2849.41 ± 41.97
Mid diameter mean (pixels)	0.47	0.54
Mid diameter 95% CI (pixels)	0.48 ± 0.01	0.56 ± 0.01
Width of body mean (pixels)	41.3	47.07
Width of body 95% CI (pixels)	41.30 ± 0.49	47.07 ± 0.58
Bounding height mean (pixels)	78.76	77.36
Bounding height 95% CI (pixels)	77.87 ± 0.61	76.30 ± 0.73
Bounding width mean (pixels)	53.25	55.79
Bounding width 95% mean (pixels)	53.25 ± 0.54	55.79 ± 0.54
Circularity mean	0.69	0.76
Circularity 95% CI	0.69 ± 0.01	0.76 ± 0.01
Ellipticity mean	1.47	1.38
Ellipticity 95% CI	1.47 ± 0.02	1.38 ± 0.02
Regularity	1.30	1.18
Regularity 95% CI	1.30 ± 0.01	1.18 ± 0.01
Elongation mean	0.19	0.15
Elongation 95% CI	0.19 ± 0.01	0.15 ± 0.01
Length of hook mean (pixels)	12.44	9.21
Length of hook 95% CI (pixels)	12.44 ± 0.45	9.21 ± 0.52

Supplementary Data 4. Comparison of sperm head nuclear morphology parameters between WT and *Teshl*-KO sperm.

Quantitative analysis of sperm head morphology performed using image-based morphometric measurements. Parameters included area, mid diameter, width, bounding height and width, circularity, ellipticity, regularity, elongation, and hook length. Data are presented as means ± 95% confidence internal (CI) of independent sperm samples from WT and *Teshl*-KO mice. Statistical comparison between WT and *Teshl*-KO groups were performed using a two tailed Student’s *t* test.

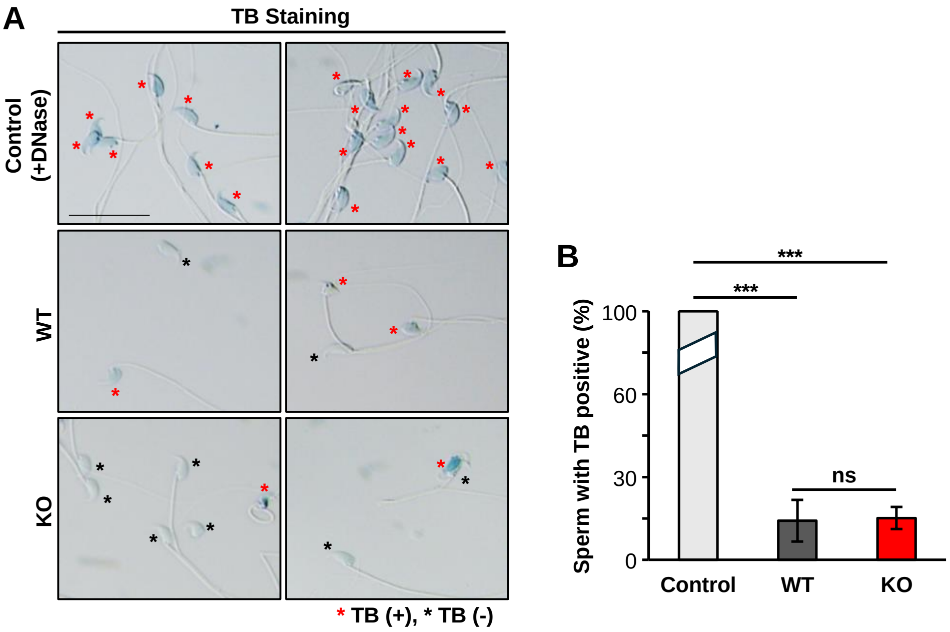

Supplementary Data 5. Toluidine blue staining analysis.
(A) Representative images of Toluidine blue (TB)-stained sperm nuclei from WT and *Teshl*-KO mice. DNase-treated sperm were used as a positive control for chromatin decondensation. Black stars, TB-negative sperm with intact chromatin; red stars, TB-positive sperm with decondensed or abnormally compacted chromatin. Scale bars: 20 μm. **(B)** Bar graph showing the percentage of TB-positive sperm. Data are presented as means ± SEM (n = 3 mice per group; $^{***}P < 0.001$, two-tailed Student's *t* test).

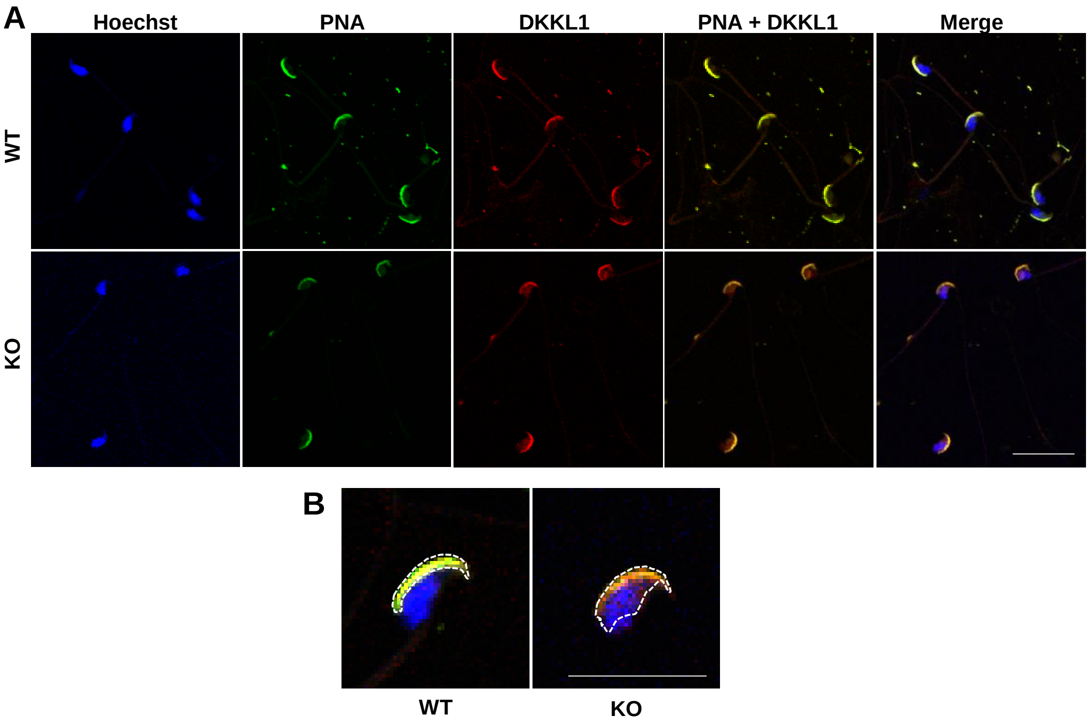

Supplementary Data 6. Localization of DKKL1.
(A) Immunofluorescence analysis showing the localization of DKKL1 in sperm from WT and *Teshl*-KO mice. Nuclei were stained with Hoechst (blue), acrosomes were stained with PNA (green), and DKKL1 was detected using a specific antibody (red). Scale bar: 12.5 μm. **(B)** Enlarged representative images highlighting DKKL1 signals in WT and *Teshl*-KO sperm. Scale bar: 2 μm.
